# Natural products against Babesiosis: active compounds, molecular mechanisms, application limitations and translational development

**DOI:** 10.3389/fcimb.2026.1921065

**Published:** 2026-08-24

**Authors:** Jianing Pang, Lixia Dai, Yang Huang, Qian Liu, Yuchao Ma, Xiaoyan Yu, Zhen Zhu, Xiaorong Yang, Xiaolou Miao, Mingze Cao, Xiaofei Shang

**Affiliations:** 1 Key Laboratory of New Animal Drug Project, Key Laboratory of Veterinary Pharmaceutical Development of Ministry of Agriculture, Lanzhou Institute of Husbandry and Pharmaceutical Sciences, Chinese Academy of Agricultural Sciences, Lanzhou, Gansu, China; 2 School of Life Sciences and Food Engineering, Hebei University of Engineering, Handan, China; 3 Department of Basic Medical Sciences, Qinghai University Medical College, Xining, China

**Keywords:** anti-Babesia agents, Babesiosis, challenge, mechanism of action, natural products

## Abstract

Babesiosis is an important tick-borne disease affecting humans and a wide range of domestic and wild animals, with substantial implications for public health, animal welfare, and livestock production. Despite the increasing global burden of babesiosis, currently available therapeutic options are limited by variable efficacy, drug resistance, adverse effects, and, in veterinary settings, concerns regarding drug residues. Natural products have emerged as an important source of antiparasitic agents owing to their remarkable chemical diversity, multi-target activities, and broad pharmacological potential. In this review, we comprehensively summarize the current knowledge on natural products with antibabesial activity, with emphasis on their major classes, representative compounds, proposed molecular mechanisms, and translational prospects, while distinguishing evidence derived from *in vitro* assays, animal studies, and the still very limited clinical investigations. Available evidence indicates that diverse natural products exhibit inhibitory activity against *Babesia* spp. predominantly *in vitro*, whereas only a smaller number have shown efficacy in animal models and clinical evidence remains scarce. These products appear to act through multiple mechanisms, such as disruption of redox balance, mitochondrial dysfunction, interference with nucleic acid biosynthesis, and suppression of energy metabolism and parasite proliferation. Although the development of natural products for babesiosis control remains hindered by insufficient mechanistic validation, limited *in vivo* and clinical evidence, poor bioavailability, safety and quality-control issues, and a lack of standardized evaluation systems, they represent promising lead resources for antibabesial drug discovery and warrant further investigation to support their translational development.

## 1 Background

The genus *Babesia* comprises intraerythrocytic protozoan parasites within the phylum Apicomplexa, including over 100 recognized species (Jerzak et al., 2023). *Babesia* transmission requires both vertebrate hosts and *ixodid ticks*, this cycle leads to hemolytic anemia and systemic inflammation, while specialized organelles, such as the apicoplast, facilitate intracellular survival and contribute to therapeutic challenges (Lemieux et al., 2016; Fuller, 2024). Approximately 20% of adult cases are subclinical infections, which pose a considerable public health risk given their potential transmission through blood transfusion (Kumar et al., 2021). Immunocompromised populations, reported case fatality rates range from 6% to 21% and may exceed 50% in severely immunocompromised patients (Aderanti et al., 2025). Globally, the major prevalent *Babesia* species include *Babesia microti*, *B. divergens*, *B. duncani*, *B. bovis*, and *B. venatorum*, and are being increasingly reported across North America, Europe, and parts of Asia (Fu et al., 2025). A detailed breakdown of the global distribution and epidemic areas for these major zoonotic *Babesia* species is presented in **Supplementary Table 1**.

Effective control measures against *Babesia* infection include tick control through acaricides, vaccination, and babesicidal drugs. Acaricides are used to target tick vectors, primarily by killing or repelling ticks to reduce the transmission of *Babesia*, but they cannot eliminate the *Babesia* pathogens from already infected ticks. Additionally, there is evidence suggesting that ticks are gradually developing resistance to them (Santos et al., 2023). Currently commercialized *Babesia* vaccines are primarily targeting bovine and canine infections. However, their widespread application is limited by safety concerns, unstable potency, high production costs, and insufficient breadth of immunoprotection. In the field of human vaccine development, a core obstacle lies in the inability to establish a long-term *in vitro* culture system for *B. microti*, this necessitates reliance on animal hosts or surrogate parasite species for antigen acquisition, significantly increasing the complexity and cost of research and development (Alzan et al., 2024). The current clinical management of babesiosis primarily relies on combination chemotherapy. While such regimens can control acute infection, they face multiple limitations: widespread drug resistance, low radical cure rates, significant adverse effects, high treatment costs, and challenges in broad implementation (Jerzak et al., 2023). Therefore, the development of novel drugs based on natural products holds considerable importance.

The medicinal use of natural products boasts a long-standing historical background. In ancient times, humans faced a wide range of various parasitic diseases and gradually identified the medicinal properties of some certain specific natural plants through sustained practice and experimentation. Ancient medical texts from North Africa, Europe, and China contain valuable records of early clinical pharmacology, including the *Ebers Papyrus* from ancient Egypt, *De Materia Medica* from ancient Greece, and *Shennong Ben Cao Jing* from China (Zhang et al., 2020). Certain classic plant species, such as the cinchona tree and *Artemisia annua*, have yielded the respective active ingredients quinine and artemisinin following long term application and screening. These compounds remain clinically utilized for the treatment of malaria in Africa and China to this day, reflecting their enduring relevance in antiparasitic therapy (Hiben et al., 2015). Natural products offer advantages such as diverse sources, structural variety, low host toxicity, and a reduced tendency to induce resistance, effectively addressing the shortcomings of existing chemical drugs.

In the following section, we discuss the importance of a better understanding of the diverse anti-*Babesia* mechanisms and structural characteristics of natural products in the context of human and animal babesiosis as a crucial tool to identify novel preventive and therapeutic agents, as well as lead compounds for drug development and resistance reversal strategies.

## 2 Current approaches to *Babesia* infection control

### 2.1 Vaccine

Vaccine research has focused mainly on bovine Babesiosis due to its high economic impact and feasibility of centralized immunization. Whole-parasite vaccines, including inactivated and attenuated live formulations, are historically established approaches. Inactivated vaccines reduce erythrocyte destruction and clinical symptoms but suffer from weak immunogenicity, reliance on adjuvants, high production costs, and complex administration (Mahoney, 1967). Attenuated live vaccines, such as splenectomized calf-derived formulations, have reduced mortality below 1% in field trials but carry contamination risks, short shelf-life, and are unsuitable for adult cattle due to potential acute disease or abortion (Callow and Mellors, 1966). Recent *in vitro*-attenuated strains, like *Att-S74-T3Bo*, bypass some safety and production issues and protect calves and adults against homologous virulent strains, yet heterologous protection and long-term immunity remain unverified (Bastos et al., 2023).

Subunit vaccines targeting specific antigens offer improved safety and controlled immune responses. In bovines, multi-antigen recombinant vaccines induce high-titer IgG and Th1 responses in murine models, inhibiting erythrocyte invasion, but efficacy in cattle is unconfirmed and some antigens, like *msa-2b*, are weakly immunogenic (Gimenez et al., 2016). Canine vaccines, such as Pirodog^®^ and Nobivac Piro^®^, use soluble parasite antigens to elicit antibody-mediated neutralization, broadening species-specific protection (Freyburger et al., 2011). Limitations include multiple doses, adjuvant dependence, technically demanding parasite cultivation, and high costs.

*Babesia* vaccines face challenges of antigenic variation, limited cross-protection, scalability, and lack of reliable tick bite challenge models. Most studies rely on blood-stage injections, ignoring tick saliva’s immunomodulatory effects (Al-Nazal et al., 2021; Ali et al., 2022). Overall, while vaccines can confer protection and remain the main preventive strategy in livestock, technical, safety, and economic constraints restrict widespread use, highlighting the continued reliance on drugs as anti-*Babesia* agents.

### 2.2 Chemical drugs and antibiotics

#### 2.2.1 Atovaquone

**Figure 1:**
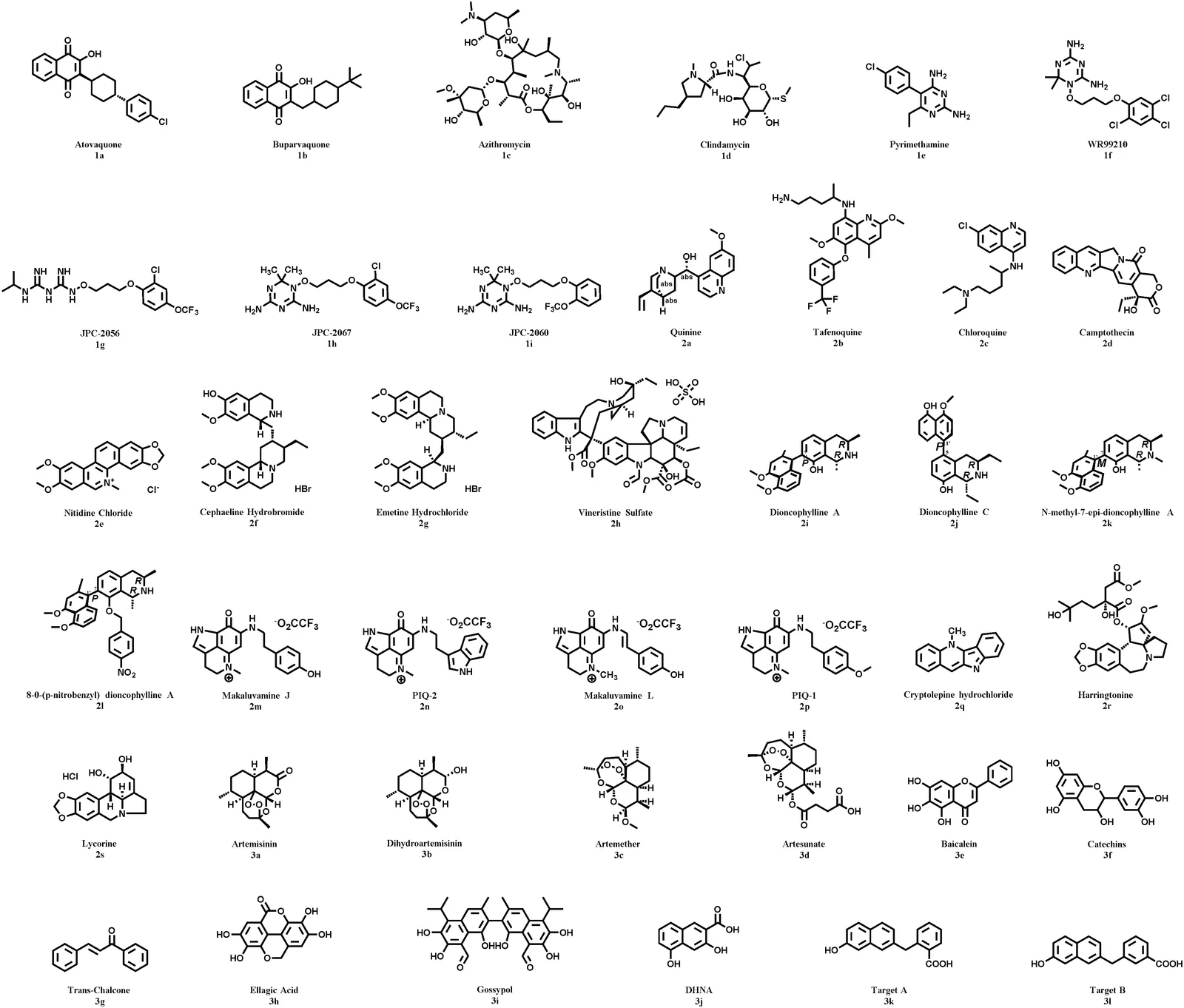
Chemical structures of compounds 1a–3l.

Atovaquone (1a) (**Figure 1**), a naphthoquinone derivative, combined with proguanil in the commercial formulation Malarone for malaria treatment (Choi et al., 2019). As a highly lipophilic compound with a relatively low molecular weight, atovaquone readily interacts with biological membranes and exerts targeted intracellular activity. In clinical, atovaquone is administered orally at a dosage of approximately 13.4 mg/kg every 8 hours. This regimen is combined with azithromycin for a total treatment duration of 10 days. Originally, atovaquone was used as monotherapy against *Pneumocystis carinii*, *Toxoplasma gondii*, and *Plasmodium* species (Bueno et al., 2012; Bonnet et al., 2023; Rodriguez and Szajnman, 2023). Compared with other antiprotozoal agents, it demonstrates an excellent safety profile. Notably, for canine *B. gibsoni* infection, atovaquone is considered the safest and most effective treatment, with minimal adverse effects (Kirk et al., 2017). Atovaquone inhibits the parasite mitochondrial electron transport chain by binding the Qo site of cytochrome bc_1_ (Vydyam et al., 2024b). This blocks ubiquinone regeneration, disrupts dihydroorotate dehydrogenase function, impairs pyrimidine biosynthesis, and leads to parasite death. Although *Babesia* and *Plasmodium* share this target, resistance evolution differs markedly. In *Plasmodium*, resistance mutations compromise mosquito-stage development, limiting transmission of resistant strains. In contrast, resistance evolution in *Babesia* exhibits distinct characteristics (Birkholtz et al., 2022; Tuvshintulga et al., 2022). Without the strict mosquito-stage bottleneck that constrains *Plasmodium*, and with the ability to establish long-term persistent infections in immunocompromised hosts, resistant mutant *Babesia* strains readily accumulate in human hosts and can spread through routes such as blood transfusion. In immunocompromised patients receiving long-term atovaquone prophylaxis or therapy, resistant *Babesia* strains carrying multiple *cytb* mutations are readily selected for, resulting in treatment failure and disease recurrence (Vydyam et al., 2024b). To mitigate resistance, atovaquone is typically used during the early to middle stages of infection, often in combination with azithromycin. While this regimen is effective for individual pets, large-scale application in animal groups or shelters is limited by high financial and logistical costs (Checa et al., 2017).

#### 2.2.2 Buparvaquone

Buparvaquone (1b), a hydroxynaphthoquinone antiprotozoal agent and first-line therapy for bovine *Theileria annulata* infection. Early studies demonstrated that doses of 2.5–6 mg/kg effectively alleviated acute symptoms in *B. equi*-infected equine models, although clearance of chronic carrier states remained variable (Zaugg and Lane, 1989). In canine infections, monotherapy often fails to achieve complete parasite elimination, necessitating combination with azithromycin to enhance therapeutic efficacy (Checa et al., 2017; Rahman et al., 2022).

Buparvaquone generally shows favorable safety and clinical tolerability during acute-phase treatment (Checa et al., 2017); however, emerging resistance poses a growing challenge. Resistance is primarily associated with mutations in the parasite *cytochrome b* gene. In *Theileria*, a single M128I substitution (methionine to isoleucine) alters the drug-binding conformation, drastically reducing buparvaquone affinity (Zaugg and Lane, 1989). In *B. gibsoni*, multi-site mutations have been reported, rendering some infections completely unresponsive to buparvaquone-azithromycin therapy (Rahman et al., 2022). Importantly, resistant strains retain the ability to proliferate and transmit, potentially establishing chronic infections (Zaugg and Lane, 1989; Tajeri et al., 2024).

#### 2.2.3 Azithromycin

In 1980, Croatian researchers first synthesized azithromycin (1c), a second-generation broad-spectrum macrolide antibiotic with a novel nitrogen-containing lactone structure, through chemical modification of erythromycin A (Oliver and Hinks, 2021). This landmark discovery not only expanded its applications in antibacterial and antiviral therapy (Qin and Ma, 2020) but also, due to its unique pharmacokinetic properties, suggested potential for antiparasitic use. Multiple studies in the 1990s confirmed azithromycin’s specific inhibitory effects on Apicomplexa protozoa (Firth and Prathapan, 2020; Jhulki et al., 2025).

Both atovaquone and azithromycin are effective as monotherapies against *Babesia* species, but their combination demonstrates superior efficacy. The atovaquone-azithromycin regimen is currently the only treatment proven to successfully clear *B. conradae* infections in dogs. A commonly employed treatment regimen involves the administration of atovaquone at a dosage of 13.5 mg/kg three times daily, combined with azithromycin at 10 mg/kg once daily, with the entire therapeutic course lasting 10 days (Ma et al., 2024). Moreover, this combination has shown high safety and therapeutic efficacy in treating the novel *Babesia* strain EBP01 infecting giant pandas, alleviating acute infections and improving anemia symptoms (Yin et al., 2022; Krause et al., 2024). However, azithromycin is associated with gastrointestinal side effects, which may increase discomfort in treated animals (Rodrigues FF. et al., 2023; Krause et al., 2024). Resistance to the atovaquone-azithromycin combination has been reported in *B. microti* infections, particularly in highly immunocompromised patients (Dunay et al., 2018). In such cases, the treatment course may need to extend to six weeks or longer to achieve clinical resolution (Ma et al., 2024).

#### 2.2.4 Clindamycin

Clindamycin (1d) is a broad-spectrum antibiotic primarily used to treat infections caused by anaerobic bacteria (Rodrigues FF. et al., 2023) and was historically applied in the management of *T*. *gondii* infections (Dunay et al., 2018). Currently, clindamycin is available mainly in two salt forms, Clindamycin hydrochloride and Clindamycin phosphate, which differ in solubility and stability, enabling versatile administration via oral or injectable routes (Shamsi et al., 2017; Lu et al., 2021; Feng et al., 2022; Ghosh et al., 2025).

The efficacy of clindamycin monotherapy varies depending on the species and route of administration. In *B. microti*-infected Mongolian gerbils, oral clindamycin at 300 mg/kg showed limited activity. In hamsters, 150 mg/kg administered orally or intramuscularly reduced peak parasitemia by approximately twofold (Renard and Ben Mamoun, 2021). In BALB/c mice, intraperitoneal injection of 500 mg/kg decreased peak parasitemia by roughly threefold (AbouLaila et al., 2012). Clindamycin is frequently combined with other agents to enhance therapeutic outcomes. The clindamycin-quinine combination remains the standard for severe babesiosis (Sanchez et al., 2016). In *B. gibsoni*-infected dogs where atovaquone-azithromycin therapy failed, a triple regimen of clindamycin, metronidazole, and doxycycline increased the PCR-negative conversion rate from 25% to 87%, highlighting the advantages of combination therapy in overcoming monotherapy limitations (Almendros et al., 2020).

Nevertheless, gastrointestinal and allergic reactions are common, limiting its combination use with quinine in clinical practice (Huang et al., 2023). Mechanistically, azithromycin and clindamycin do not directly induce immediate parasite death; instead, they lead to the production of daughter cells that are incapable of subsequent division, resulting in delayed clinical resolution after treatment (Capasso and Supuran, 2023).

#### 2.2.5 Antifolate agents

Folate metabolism is an essential and evolutionarily conserved pathway in apicomplexan parasites, providing one-carbon cofactors required for purine, pyrimidine, and amino acid biosynthesis and thereby supporting DNA replication and parasite proliferation. The bifunctional enzyme dihydrofolate reductase-thymidylate synthase (DHFR-TS), which catalyzes both the NADPH-dependent reduction of dihydrofolate to tetrahydrofolate and the methylation of deoxyuridine monophosphate to deoxythymidine monophosphate, is the principal target of most antifolate drugs (Aboge et al., 2008). Because protozoan DHFR-TS differs substantially from the human ortholog, antifolates display selective antiparasitic activity and have been extensively investigated for babesiosis (Begley et al., 2011; Vydyam and Ben Mamoun, 2026a).

Pyrimethamine (1e), a prototypical first-generation diaminopyrimidine DHFR inhibitor, shows marked interspecies variation in anti-babesial activity. In *B. bovis*, it suppresses parasite growth but is less potent than diminazene aceturate, while exhibiting no detectable cytotoxicity to mammalian cells over a wide concentration range (Vydyam et al., 2024a). Its only moderate efficacy has been attributed to naturally occurring polymorphisms in the *B. bovis dhfr-ts* gene that alter the DHFR binding pocket (Begley et al., 2011). Among equine *Babesia* species, *B. equi* is less susceptible to pyrimethamine than *B. caballi*, and viability assays indicate irreversible killing in *B. equi* at inhibitory concentrations, whereas *B. caballi* can regrow after drug withdrawal (Nagai et al., 2003). In human-pathogenic species, pyrimethamine shows moderate activity against *B. duncani* with acceptable selectivity in human cell lines, but demonstrates limited efficacy in *B. microti*-infected rodent models, highlighting substantial heterogeneity in antifolate susceptibility across the genus (Vydyam et al., 2024a).

Second-generation dihydrotriazines were developed to address the limited potency of pyrimethamine. WR99210 (1f) displays markedly enhanced activity against *B. duncani*, with potency far exceeding that of pyrimethamine, and directly inhibits recombinant DHFR-TS from both *B. duncani* and *B. microti*. It also exhibits minimal cytotoxicity in multiple human cell lines, indicating a very favorable therapeutic window (Singh et al., 2023). Nevertheless, its further development has been constrained by poor oral bioavailability and gastrointestinal toxicity.

Third-generation biguanide prodrugs were therefore designed to improve pharmacokinetics through hepatic conversion to active dihydrotriazines. JPC-2056 (1g) and its metabolite JPC-2067 (1h) showed potent activity against *B. duncani*, and JPC-2056 significantly delayed parasitemia and improved survival in infected mice, while completely clearing *B. microti* in SCID mice without recrudescence. Subsequent screening identified several broad-spectrum dihydrotriazine and biguanide analogs with sub-10 nM activity against multiple *Babesia* species. Notably, JPC-2060 (1i) displayed uniformly high potency and excellent selectivity (Vydyam et al., 2024a; Vydyam and Ben Mamoun, 2026a). Collectively, these findings establish antifolates as a promising class for babesiosis, although further optimization of oral exposure, *in vivo* efficacy, and combination strategies will be necessary for clinical translation.

At present, the chemotherapeutic agents clinically used for the treatment of babesiosis, when administered long-term as monotherapy or used inappropriately in combination, have induced specific gene mutations associated with drug resistance in *Babesia*, thereby reducing drug efficacy and leading to chronic persistent infection. The resistance-associated mutation sites, mutation types, and resistance phenotypes of *Babesia* corresponding to first-line drugs such as atovaquone, buparvaquone, and azithromycin are shown in **Table 1**.

**Table 1:** Clinical anti-Babesia drugs and related Babesia resistance mutations.

Drug	Babesia species	Zoonotic (yes/no/unknown)	Protein target	Reported mutations or variants	Type of evidence source	Clinical relevance (yes/no/unknown)	Ref.
Atovaquone	*B. microti*	yes	CYTb	Y272C	human clinical	yes	(Simon et al., 2017; Marcos and Wormser, 2023)
Atovaquone	*B. microti*	yes	CYTb	Y272S	human clinical	yes	(Holbrook et al., 2023)
Atovaquone	*B. microti*	yes	CYTb	M134I	human clinical	yes	(Holbrook et al., 2023) (Marcos and Wormser, 2023)
Atovaquone	*B. microti*	yes	CYTb	M134T	human clinical	yes	(Holbrook et al., 2023) (Marcos and Wormser, 2023)
Atovaquone	*B. microti*	yes	CYTb	V141A	human clinical	yes	(Rogers et al., 2023) (Holbrook et al., 2023)
Atovaquone	*B. microti*	yes	CYTb	L277P	human clinical	yes	(Marcos and Wormser, 2023)
Atovaquone	*B. microti*	yes	CYTb	L334I	human clinical	unknown	(Holbrook et al., 2023)
Atovaquone	*B. gibsoni;*	unknown	CYTb	M121I	dog clinical/*in vitro*	no	(Iguchi et al., 2013; Liu et al., 2016)
Azithromycin	*B. microti*	yes	RPL4	R86C	human clinical	yes	(Simon et al., 2017)
Azithromycin	*B. microti*	yes	RPL4	R86H, S73L, C103Y, S121G	human clinical	unknown	(Holbrook et al., 2023; Marcos and Wormser, 2023)
Azithromycin + Clindamycin	*B. microti*	yes	23S rRNA V	A1915G	human clinical	yes	(Rogers et al., 2023)
Diminazene aceturate	*B. bovis*	no	unknown	Unknown	*in vitro*	no	(Tuvshintulga et al., 2019)
Clindamycin	*Babesia* spp.	yes	unknown	Unknown	human clinical	unknown	(Rogers et al., 2023)

## 3 Natural products and derivatives against *Babesia* infection diseases

### 3.1 Alkaloids and their derivatives

#### 3.1.1 Quinoline compounds

Quinine (2a), also known as a cinchona alkaloid, is a representative natural quinoline compound derived from the bark of the cinchona tree. Historically, its medicinal value dates back to the 17th century and it has long been used as an antimalarial agent (Akhtar et al., 2020). Industrial formulations, such as quinine sulfate tablets and quinine dihydrochloride injections, replaced the crude use of cinchona bark and became core treatments for malaria (Liang et al., 2023). In May 1982, the first successful clinical use of quinine for babesiosis was reported, highlighting its antiparasitic potential. Mechanistically, studies suggest quinine may target purine nucleoside phosphorylase (Dziekan et al., 2019) and interfere with parasite membrane structures, including the plasma membrane, endoplasmic reticulum, and mitochondria (Renard and Ben Mamoun, 2021).

Based on the structure of quinine, scientists have successfully synthesized many safer and more effective analogs. Among them, Tafenoquine (2d) is an important achievement and one of the most promising anti-*Babesia* agents of this series of drugs. In experimental models of human babesiosis, tafenoquine showed broad activity against multiple zoonotic species, including *B. microti*, *B. duncani*, *B. divergens* Notably, preclinical studies demonstrated that tafenoquine combined with atovaquone can achieve radical cure and even sterile immunity, preventing recrudescence more effectively than monotherapy (Vydyam et al., 2024b).

Tafenoquine is also highly relevant clinically, particularly for relapsing human babesiosis in immunocompromised patients, such as those receiving rituximab or chemotherapy, in whom persistent infection and resistance to standard regimens are common. Recent U.S. case reports and case series suggest that tafenoquine-containing regimens can help clear persistent parasitemia and support sustained PCR negativity in refractory cases, although monotherapy may be insufficient in some patients (Rogers et al., 2023).

From a U.S. translational perspective, the commercial tafenoquine formulation ARAKODA is of particular importance. Already approved for malaria prevention, it has received FDA orphan drug designation for acute babesiosis, highlighting its growing therapeutic significance (Krause et al., 2024) (Sixty Degrees Pharmaceuticals, 2025). In addition, its long plasma half-life (approximately 14–28 days) enables simplified loading plus weekly maintenance dosing (Liu et al., 2021), offering practical advantages for long-term management despite the requirement for prior G6PD screening (Rogers et al., 2023).

Although quinine demonstrates strong *in vitro* antiparasitic activity, monotherapy has limited clinical efficacy (Lawres et al., 2016; Zhang et al., 2021). Clinically, it is usually combined with clindamycin, forming the first effective antimicrobial therapy for babesiosis and a classic treatment regimen prior to the introduction of atovaquone-azithromycin (Smith et al., 2020; Huang et al., 2023; Fu et al., 2025). However, this combination is associated with significant adverse effects—including nausea, vomiting, tinnitus, hearing loss, blurred vision, and in severe cases, arrhythmia or hypotension-limiting its broad clinical application. Moreover, mortality rates with clindamycin-quinine are higher than with atovaquone-azithromycin, relegating it to an alternative therapy when first-line treatment fails (Kletsova et al., 2017).

Chloroquine (2c), a low-cost and relatively safe antimalarial particularly when combined with iron chelators or artemether, has demonstrated *in vitro* activity against *B. bovis*, indicating potential cross-species efficacy (Ferrer et al., 2012; Koonyosying et al., 2023). However, therapeutic outcomes vary among *Babesia* species. This variability may be due to the absence of a digestive vacuole, preventing chloroquine from acting via the classic “heme capture” mechanism observed in *Plasmodium*; its observed effects likely result from interference with parasite nucleic acid synthesis (Galappaththy et al., 2013).

Camptothecin (2d), a topoisomerase I inhibitor initially developed for anticancer application, has shown promising activity against *Babesia* and *Theileria* species due to shared apicomplexan biology. It inhibits *B. bigemina*, and at higher concentrations prevents parasite regrowth, suggesting potential for complete parasite clearance in clinical settings (Tayebwa et al., 2018).

#### 3.1.2 Isoquinoline compounds

Isoquinoline alkaloids are natural compounds featuring a fused benzene–pyridine ring system. This structure allows diverse chemical modifications and strong interactions with cellular targets, giving them broad antiparasitic activity and low toxicity to host cells. Several isoquinoline derivatives show promising activity against *Babesia* species. Nitidine chloride (2e) from *Zanthoxylum nitidum* inhibits *B. bovis*, *B. bigemina*, and *B. caballi*, causing parasite deformation and debris accumulation in red blood cells. *In vivo*, nitidine chloride reduces parasitemia in *B. microti*-infected mice by over 80%, although PCR indicates potential residual infection (Tayebwa et al., 2018). Other plant-derived isoquinolines, including cephaeline hydrobromide (2f), emetine hydrochloride (2g), and vincristine sulfate (2h), show strong anti-*B. gibsoni* activity and can irreversibly disrupt parasite morphology without harming host cells (Ji et al., 2021).

Naphthylisoquinoline alkaloids from tropical lianas, such as dioncophylline A (2i), dioncophylline C (2j), and N-methyl-7-epi-dioncophylline A (2k), also display significant anti-*Babesia* activity. Synthetic derivatives, like 8-O-(p-nitrobenzyl) dioncophylline A (2l), show potential for pharmaceutical development. Notably, some isoquinoline compounds effective against *Plasmodium* are weak against *Babesia*, suggesting species-specific mechanisms (Bringmann et al., 2020).

#### 3.1.3 Other alkaloids

Quinn and colleagues (Davis et al., 2012) systematically isolated a series of pyrroloiminoquinone (PIQ) alkaloids from the Australian marine sponge *Zyzzya* sp. in 2012, marking a breakthrough in antimalarial research. Several PIQs exhibited potent antimalarial activity with significantly reduced cytotoxicity toward HEK293 cells, demonstrating strong application potential. Subsequent studies optimized synthetic strategies to produce 16 natural PIQs and structural analogs, revealing robust inhibitory activity against *Babesia*. Notably, imine nitrogen-methylated makaluvamine-type compounds, including makaluvamine J (2m), PIQ-2 (2n), makaluvamine L (2o), and PIQ-1 (2p), showed very low IC_50_ values against *B. divergens* Rouen. PIQ-2, in particular, exhibited an IC_50_ below 2 nM and a therapeutic index (TI) of 154.41, substantially surpassing traditional combination therapies (Barnes et al., 2024).

Cryptolepine hydrochloride (2q), the primary indoloquinoline alkaloid from the roots of *Cryptolepis sanguinolenta*, a shrub widely used in Central and West African traditional medicine, has long been applied to treat febrile illnesses, including malaria, hepatitis, and bacterial infections (Forkuo et al., 2016). It strongly inhibits multiple *Babesia* species, with IC_50_ values substantially lower than positive controls (Batiha et al., 2020). *In vivo*, half-dose cryptolepine hydrochloride combined with atovaquone or diminazene aceturate achieves chemotherapeutic effects comparable to full-dose monotherapy, effectively suppressing *B. microti* proliferation. Furthermore, combination therapy accelerates parasite clearance. However, cryptolepine hydrochloride-diminazene aceturate demonstrates moderate antagonism against *B. bigemina* and *B. divergens*, suggesting species-specific efficacy that warrants further mechanistic investigation (Batiha et al., 2020; Zhang et al., 2021).

Additionally, harringtonine (2r) and lycorine (2s) exhibit promising anti-*Babesia* activity. Both compounds exhibit extremely low cytotoxicity toward mammalian cells, with selectivity indices of 49,670.9 for harringtonine and 1,639.1 for lycorine, allowing for safe use even at relatively high concentrations. Notably, treatment at 2×IC_50_ disrupts typical parasite morphology without damaging canine red blood cells, and 4×IC_50_ prevents parasite regrowth after transfer to drug-free medium, indicating reliable killing capacity. Although harringtonine is known to inhibit the initial elongation step of protein synthesis by interacting with the ribosomal A-site, whether this mechanism applies to *B. gibsoni* remains to be confirmed (Ji et al., 2021).

Collectively, these marine- and plant-derived natural products exhibit low toxicity, high selectivity, and potent inhibitory activity against *Babesia*, representing promising candidates for drug development. However, single-agent efficacy appears species-dependent, and complete parasite clearance requires further validation. Future studies integrating mechanistic insights and combination therapies are needed to facilitate clinical translation.

### 3.2 Sesquiterpenes

#### 3.2.1 Artemisinin

Artemisinin (3a) is an active compound extracted from *Artemisia annua*, a plant used in traditional Chinese medicine for malaria treatment since the mid-4th century, as recorded in *The Handbook of Prescriptions for Emergencies*. Early experiments using water or ethanol extracts failed to demonstrate consistent therapeutic effects (Dong et al., 2022). A major breakthrough came in 1971 through China’s 523 Project, led by Tu Youyou, which successfully isolated an active ingredient from *A. annua* and named it artemisinin, which achieved 100% parasite clearance in malaria animal models, including *P. berghei* and *P. cynomolgi*, opening a new era in antimalarial therapy (Ma et al., 2020). *In vitro* studies targeting *B. duncani* have confirmed that artemisinin exhibits inhibitory activity, with an IC_50_ of 14 μM, surpassing that of clindamycin, a commonly used clinical drug (Zhang et al., 2021). Moreover, a 30% ethanol extract of *A. annua* showed superior activity compared to higher-concentration ethanol extracts, suggesting that plant components such as flavonoids may synergize with artemisinin to enhance efficacy (Si et al., 2023).

However, the streamlined genome of *Babesia* lacks the heme-utilization mechanisms found in *Plasmodium*, reducing the parasite’s sensitivity to artemisinin (Si et al., 2023). Sub-culture experiments further revealed that parasites treated with artemisinin could relapse after drug withdrawal at concentrations ranging from 1× to 4×IC_50_, indicating that its parasiticidal effect is insufficient for complete clearance of infection (Zhang et al., 2021).

#### 3.2.2 Artemisinin derivatives

The unique endoperoxide (peroxide) bridge of artemisinin and the heme released by *Plasmodium* are key contributors to its antimalarial activity (Loo et al., 2017). However, compared to *Plasmodium*, *Babesia* has a smaller genome and lacks the enzymes required for complete heme biosynthesis. This suggests that *Babesia* cannot synthesize heme *de novo*, making it less sensitive to artemisinin (Si et al., 2023). Consequently, researchers have turned to evaluating artemisinin derivatives for enhanced efficacy.

Dihydroartemisinin (3b) is a reduced hemiacetal derivative of artemisinin and a major active metabolite with improved bioavailability and more stable pharmacokinetics. *In vitro* studies show strong growth-inhibitory activity against *B. gibsoni*, with an IC_50_ of 0.938 μM, indicating potent antiparasitic activity. Despite its promising *in vitro* performance, there is currently insufficient *in vivo* evidence to support complete parasite clearance with monotherapy (Matsuu et al., 2008).

Artemether (3c) exhibits notable *in vitro* efficacy, with lower IC_50_ values and higher killing efficiency compared to quinine and clindamycin. Nevertheless, practical application is limited: complete inhibition of parasite regrowth requires relatively high concentrations, and sensitivity varies among *Babesia* species (Zhang et al., 2021). Moreover, artemether has poor pharmacokinetics, including a short half-life that prevents sustained therapeutic concentrations *in vivo*. Its main advantage lies in strong synergism with lumefantrine, suggesting that combination therapy could significantly enhance antiparasitic effects through a dual-target mechanism (Iguchi et al., 2015).

Artesunate (3d) demonstrates superior single-agent activity compared to artemether. *In vitro*, it inhibits wild-type *B. gibsoni* with an IC_50_ of 0.878 µM, compared to 4.72 µM for artemether (Matsuu et al., 2008). In a *B. microti* mouse model, artesunate not only inhibited parasite growth but also delayed the rise of parasitemia (Loo et al., 2017). Additionally, artesunate treatment mitigated inflammatory liver damage in a dose-dependent manner, preserving hepatic lobule structure and central vein integrity at high doses (8 mg/kg) (Fazilani et al., 2024). Despite these benefits, artesunate alone fails to eradicate parasites, as parasitemia rebounded after treatment cessation (Loo et al., 2017). Currently, systematic studies on the synergistic potential of artesunate or artemether with existing antiparasitic drugs are lacking, and the precise mechanisms of action against *Babesia* remain to be fully elucidated.

### 3.3 Flavonoids

#### 3.3.1 Baicalein

Baicalein (3e) is a major flavonoid extracted from the roots of the traditional Chinese medicinal herb *Scutellaria baicalensis* and represents one of the key pharmacologically active components of the plant. *In vitro* studies have demonstrated that baicalein exhibits definite inhibitory activity against *B. duncani*, with an IC_50_ of 12 μM. Its efficacy is significantly greater than that of clindamycin and comparable to quinine, highlighting its potential as a monotherapy candidate (Zhang et al., 2021). In addition, baicalein possesses anti-inflammatory and antioxidant properties, which may mitigate histopathological damage caused by infection and provide synergistic therapeutic benefits (Pang et al., 2016).

Animal models and preclinical studies indicate that baicalein is well-tolerated, with a safe daily dose range of 200–800 mg (Pang et al., 2016). However, sub-culture experiments suggest that baicalein may inhibit parasite growth without completely eliminating the infection: even at 8× IC_50_ concentrations, parasites can recur after drug withdrawal, indicating a risk of post-treatment relapse (Zhang et al., 2021). Currently, the potential synergistic effects of baicalein in combination with established anti-*Babesia* drugs have not been fully explored, and its pharmacokinetic properties require optimization. Further studies are needed to investigate combination therapy strategies and clarify its mechanism of action against *Babesia*.

#### 3.3.2 Catechins

Catechins (3f), first extracted from tea leaves in the early 20th century, are the main polyphenolic components of tea and contribute to its characteristic bitter taste (Li X. et al., 2022). Catechin hydrate has demonstrated significant inhibitory activity against *Babesia gibsoni*, with an IC_50_ of 273 nM, surpassing the commonly used drug diminazene aceturate, highlighting its strong efficacy as a single-agent treatment. In canine models infected with *B. gibsoni*, catechin hydrate at a dose of 11 mg/kg effectively suppressed parasite growth and improved anemia-related hematological parameters, with results comparable to the positive control drug (Zhai et al., 2022). However, catechins do not completely eradicate the parasites; post-treatment blood smears still show 0–1 parasites per field, and hematological indices do not fully return to normal. The potential synergistic effects of catechins with other anti-*Babesia* drugs have not been systematically evaluated, and the precise mechanisms underlying their antiparasitic activity remain to be elucidated (Zhai et al., 2022).

#### 3.3.3 *Trans*-chalcone

Trans-chalcone (3g), a key intermediate in plant flavonoid biosynthesis, is widely present in various plants (Asquith et al., 2025). It exhibits significant activity against babesiosis, showing inhibitory effects *in vitro* against *B. bovis*, *B. bigemina*, *B. divergens*, and *B. caballi*, with IC_50_ values of 69.6, 33.3, 64.8, and 18.9 μM, respectively, without adversely affecting host erythrocyte function (Batiha et al., 2019). *In vivo*, intraperitoneal administration at 25 mg/kg reduced peak parasitemia by 71.8% in mice infected with *B. microti*. Notably, combination therapy with diminazene aceturate and clofazimine showed superior efficacy compared to monotherapy, with no parasitic DNA detectable 45 days post-infection (Batiha et al., 2019). It also demonstrates low cytotoxicity to host cells. However, its efficacy as a monotherapy *in vivo* is lower than that of existing drugs, and the precise anti-parasitic mechanism remains unclear. It has been speculated that trans-chalcone may target the parasite’s mitochondrial respiratory chain, but this hypothesis has not yet been experimentally validated in *Babesia* species.

### 3.4 Polyphenol

#### 3.4.1 Ellagic acid

Ellagic acid (3h) is a polyphenolic compound naturally present in various plants and has demonstrated research potential in anti-parasitic applications (Zheng et al., 2025). Ellagic acid has been proven to significantly inhibit the growth of various *Babesia* species *in vitro* studies. However, its direct application is limited by insufficient chemical stability, necessitating the use of novel drug delivery systems as carriers to ensure the effective exertion of its anti-parasitic activity. Nano-formulations of ellagic acid, including β-cyclodextrin ellagic acid (β-CD EA) and ellagic acid prepared by antisolvent precipitation with a syringe pump (APSP EA), show promising potential for the treatment of babesiosis (Rodrigues MP. et al., 2023). *In vitro* studies have confirmed that all three formulations significantly inhibit the growth of multiple *Babesia* species, including *B. bovis* and *B. caballi*. Notably, APSP EA combination therapy with atovaquone resulted in undetectable parasitic DNA by day 42 post-infection, demonstrating both inhibitory and parasiticidal effects (Beshbishy et al., 2019).

#### 3.4.2 Gossypol and its derivatives

Gossypol (3i) was named gossypol due to its origin from the genus *Gossypium* and its polyphenolic chemical nature (Tang et al., 2024). *In vitro* studies have shown that the concentration of gossypol required to completely inhibit the growth of *B. bigemina* is similar to that needed for *B. bovis* (He et al., 2022). In regions with high prevalence of bovine babesiosis, co-infections with *B. bovis* and *B. bigemina* are common, complicating disease management. Administering similar dosages of gossypol can simultaneously suppress the proliferation and transmission of both pathogens, offering a practical strategy for integrated disease control (Teel and Hairgrove, 2024). However, gossypol’s toxicity remains a major concern. Monogastric animals and young ruminants are particularly susceptible, exhibiting respiratory distress, stunted growth, anemia, and thyroid metabolism disruptions upon exposure (Yu et al., 2024). Additionally, gossypol adversely affects reproductive and immune system function, further limiting its *in vivo* utility (Li WJ. et al., 2022; Pang et al., 2022).

Encouragingly, gossypol derivatives with improved safety profiles have emerged. The derivative ST087010, synthesized in 2022, exhibited low toxicity while demonstrating potent inhibitory effects against the Zika and Dengue viruses (Gao et al., 2022), suggesting the potential to develop gossypol analogs with both high antiparasitic activity and safety. Based on the finding that gossypol effectively inhibits *Babesia* lactate dehydrogenase (LDH), researchers screened three naphthalene compounds (NDCA, DBHCA, DHNA) from a gossypol-like compound library and selected DHNA (3,5-dihydroxy-2-naphthoic acid) (3j) as the lead compound due to its superior activity (Yu et al., 2020). Using computer-aided drug design, modifications of DHNA’s carboxyl and hydroxyl groups generated a derivative library through molecular docking. Two compounds target A (TA) (3k) and target B (TB) (3l) were identified. Both bind to and inhibit *B. microti* LDH, effectively suppressing parasite growth *in vitro*. TB shows stronger inhibitory activity than TA, with higher potency against BmLDH and more pronounced *in vitro* suppression of *B. microti*. Conversely, TA demonstrates better safety, exhibiting lower cytotoxicity *in vitro* cells and a higher selectivity index (SI), suggesting superior therapeutic potential. Although surface plasmon resonance studies revealed that TA and TB have over threefold higher binding affinity to BmLDH compared to DHNA, their inhibitory effects on enzyme activity and parasite growth were weaker than DHNA. This discrepancy is likely due to rapid binding but fast dissociation of TA and TB from the target, resulting in shorter-lasting effects (Luo et al., 2025).

Drug combination strategies are summarized in **Table 2**, and a comparative summary of natural products with anti-Babesia activity is presented in **Table 3**.

**Table 2:** Natural products and their derivatives–drug combination strategies against parasitic infections.

Natural product	Source	Combined drug	Target parasite	Host	Model (*in vitro*/*in vivo*)	Ref.
Quinine	*Cinchona* spp.	Clindamycin	*B. microti*/*B. divergens*/*B. venatorum*/*B. duncani*	mouse/human	*in vivo*	(Kletsova et al., 2017; Smith et al., 2020; Huang et al., 2023; Fu et al., 2025)
Artemether	Artemisinin derivatives	Lumefantrine	*B. gibsoni*	dog	*in vitro*	(Iguchi et al., 2015)
Cryptolepine hydrochloride	*Cryptolepis sanguinolenta*	Atovaquone	*B. bovis*/*B. bigemina*/*B. divergens*/*B. caballi*	cattle/horse	*in vitro*	(Batiha et al., 2020)
			*B. microti*	mouse	*in vivo*	
		Diminazene aceturate	*B. bovis*/*B. bigemina*/*B. divergens*/*B. caballi*	cattle/horse	*in vitro*	
		Clofazimine	*B. bovis*/*B. bigemina*/*B. divergens*/*B. caballi*	cattle/horse	*in vitro*	
*Trans*-chalcone	Various plants	Atovaquone	*B. bovis*/*B. bigemina*/*B. divergens*/*B. caballi*	cattle/horse	*in vitro*	(Batiha et al., 2019)
			*B. microti*	mouse	*in vivo*	
		Diminazene aceturate	*B. bovis*/*B. bigemina*/*B. divergens*/*B. caballi*	cattle/horse	*in vitro*	
			*B. microti*	mouse	*in vivo*	
		Clofazimine	*B. bovis*/*B. bigemina*/*B. divergens*/*B. caballi*	cattle/horse	*in vitro*	
			*B. microti*	mouse	*in vivo*	
APSP EA	Various plants	Atovaquone	*B. bovis*/*B. bigemina*/*B. divergens*/*B. caballi*	cattle/horse	*in vitro*	(Beshbishy et al., 2019)
			*B. microti*	mouse	*in vitro*	
		Diminazene aceturate	*B. bovis*/*B. bigemina*/*B. divergens*/*B. caballi*	cattle/horse	*in vitro*	
			*B. microti*	mouse	*in vitro*	
		Clofazimine	*B. bovis*/*B. bigemina*/*B. divergens*/*B. caballi*	cattle/horse	*in vitro*	
			*B. microti*	mouse	*in vitro*	

**Table 3:** Natural products and their derivatives with anti-Babesia activity.

Compounds	Species(strain)	Level of evidence (*in vitro*/*in vivo*)	Effect (IC_50_/inhibition rate)	Positive control	Positive control effect (IC_50_/inhibition rate)	Ref.
Quinine	*B. duncani*	*In vitro*	10 μM	clindamycin	37 μM	(Zhang et al., 2021)
Chloroquine	*B. bovis*	*In vitro*	2.14 ± 0.76 μM	diminazine aceturate	0.42 ± 0.01 μM	(Koonyosying et al., 2023)
Camptothecin	*B. bovis*	*In vitro*	11.67 ± 1.6 μM	diminazene aceturate	1.96 ± 0.34 μM	(Tayebwa et al., 2018)
	*B. bigemina*	*In vitro*	4.00 ± 1.0 μM		0.39 ± 0.03 μM	
	*B. caballi*	*In vitro*	2.07 ± 0.6 μM		0.01 ± 0.004 μM	
Nitidine Chloride	*B. bovis*	*In vitro*	1.01 ± 0.2 μM	diminazene aceturate	1.96 ± 0.34 μM	
	*B. bigemina*	*In vitro*	5.34 ± 1.0 μM		0.39 ± 0.03 μM	
	*B. caballi*	*In vitro*	0.11 ± 0.03 μM		0.01 ± 0.004 μM	
	*B. microti*	*In vivo*	90.7% (i.p.); 83.6% (p.o.)		93.7% (i.p.)	
cephaeline hydrobromide	*B. gibsoni*	*In vitro*	108.1 ± 4.3 nM	diminazene aceturate	11.6 ± 1.1 nM	(Ji et al., 2021)
emetine hydrochloride	*B. gibsoni*	*In vitro*	253.1 ± 1.4 nM	diminazene aceturate	11.6 ± 1.1 nM	
vincristine sulfate	*B. gibsoni*	*In vitro*	643.0 ± 2.8 nM	diminazene aceturate	11.6 ± 1.1 nM	
dioncophylline A	*B. canis*	*In vitro*	0.48 ± 0.03 μM	Imidocarb dipropionate	0.07 ± 0.004 μM	(Bringmann et al., 2020)
dioncophylline C	*B. canis*	*In vitro*	0.85 ± 0.04 μM		0.07 ± 0.004 μM	
N-methyl-7-epi-dioncophylline A	*B. canis*	*In vitro*	0.14 ± 0.01 μM		0.07 ± 0.004 μM	
8-O-(p-nitrobenzyl) dioncophylline A	*B. canis*	*In vitro*	0.82 ± 0.04 μM		0.07 ± 0.004 μM	
makaluvamine J	*B. divergens*	*In vitro*	3.5 nM	—	—	(Barnes et al., 2024)
	*B. duncani*	*In vitro*	166.1 nM	—	—	
PIQ-2	*B. divergens*	*In vitro*	1.6 nM	—	—	
	*B. duncani*	*In vitro*	30.1 nM	—	—	
makaluvamine L	*B. divergens*	*In vitro*	56.3 nM	—	—	
	*B. duncani*	*In vitro*	207.5 nM	—	—	
PIQ-1	*B. divergens*	*In vitro*	3.5 nM	—	—	
	*B. duncani*	*In vitro*	68.4 nM	—	—	
Cryptolepine hydrochloride	*B. bovis*	*In vitro*	1740 ± 0.37 nM	clofazimine	8240 ± 1.7 nM	(Batiha et al., 2020)
	*B. bigemina*	*In vitro*	1400 ± 0.6 nM		5730 ± 1.9 nM	
	*B. divergens*	*In vitro*	790 ± 0.32 nM		13850 ± 4.3 nM	
	*B. caballi*	*In vitro*	600 ± 0.53 nM		7950 ± 1.8 nM	
	*B.microti*	*In vivo*	79.4% (i.p.)	diminazene aceturate	86.4% (i.p.)	
				atovaquone	85.6% (i.p.)	
harringtonine	*B. gibsoni*	*In vitro*	23.4 ± 1.2 nM	diminazene aceturate	11.6 ± 1.1 nM	(Ji et al., 2021)
lycorine	*B. gibsoni*	*In vitro*	784.4 ± 3.3 nM	diminazene aceturate	11.6 ± 1.1 nM	
Artemisinin	*B. duncani*	*In vitro*	14 μM	clindamycin	37 μM	(Zhang et al., 2021)
Dihydroartemisinin	*B. gibsoni*	*In vitro*	937.50 ± 45.21 nM	—	—	(Matsuu et al., 2008)
Artemether	*B. duncani*	*In vitro*	7.8 μM	clindamycin	37 μM	(Zhang et al., 2021)
Artesunate	*B. gibsoni*	*In vitro*	878.89 ± 27.13 nM	—	—	(Matsuu et al., 2008)
Baicalein	*B. duncani*	*In vitro*	12 μM	clindamycin	37 μM	(Zhang et al., 2021)
Catechins	*B. gibsoni*	*In vitro*	273 nM	Diminazene aceturate	449 nM	(Zhai et al., 2022)
*Trans-*Chalcone	*B. bovis*	*In vitro*	69.6 ± 2.3 μM	Diminazene aceturate	0.35 μM	(Batiha et al., 2019)
	*B. bigemina*	*In vitro*	33.3 ± 1.2 μM		0.68 μM	
	*B. divergens*	*In vitro*	64.8 ± 2.5 μM		0.43 μM	
	*B. caballi*	*In vitro*	18.9 ± 1.7 μM		0.022 μM	
	*B. microti*	*In vivo*	71.8% (i.p.)		95.5% (i.p.)	
Ellagic acid	*B. bovis*	*In vitro*	9.6 ± 1.5 μM	Diminazene aceturate	0.35 ± 0.06 μM	(Beshbishy et al., 2019)
				Atovaquone	0.039 ± 0.002 μM	
				Clofazimine	8.24 ± 1.7 μM	
	*B. bigemina*	*In vitro*	7.9 ± 5.8 μM	Diminazene aceturate	0.68 ± 0.09 μM	
				Atovaquone	0.701 ± 0.04 μM	
				Clofazimine	5.73 ± 1.9 μM	
	*B. divergens*	*In vitro*	5.4 ± 2.8 μM	Diminazene aceturate	0.43 ± 0.05 μM	
				Atovaquone	0.038 ± 0.002 μM	
				Clofazimine	13.85 ± 4.3 μM	
	*B. caballi*	*In vitro*	3.3 ± 0.4 μM	Diminazene aceturate	0.022 ± 0.0002 μM	
				Atovaquone	0.102 ± 0.0141 μM	
				Clofazimine	7.95 ± 1.8 μM	
	*B. microti*	*In vivo*	48.9% (i.p.)	Diminazene aceturate	91.0% (i.p.)	
Gossypol	*B. bigemina*	*In vitro*	43.97 mM	Diminazene aceturate	—	(He et al., 2022)
3,5-dihydroxy 2-napthoic acid (DHNA)	*B.microti*	*In vitro*	85.65 ± 7.23 μM	Diminazene aceturate	—	(Yu et al., 2020)
target A (TA)	*B. microti*	*In vitro*	121.7 μM	Diminazene aceturate	—	(Luo et al., 2025)
target B (TB)	*B. microti*	*In vitro*	111.7 μM	Diminazene aceturate	—	

Direct cross-study quantitative comparisons are precluded by significant methodological heterogeneity among the included studies.

## 4 The potential mechanism of action of some promising natural products

### 4.1 Inhibition of energy metabolism

**Figure 2:**
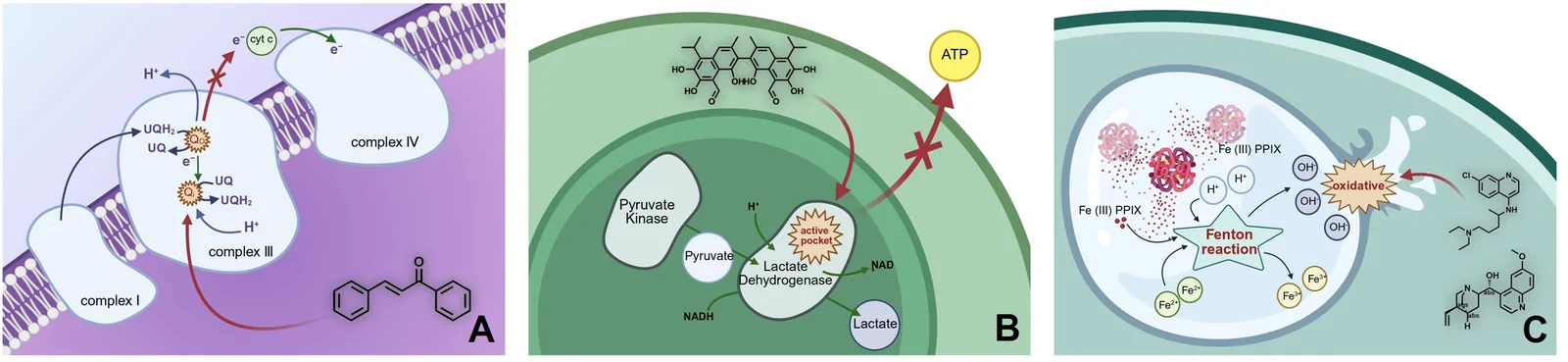
**(A)** Schematic diagram of mitochondrial complex III inhibition-mediated disruption of *Babesia* energy metabolism. Reduced ubiquinone (UQH_2_) transfers electrons to complex III, which mediates electron transfer to *cytochrome c* (*cyt c*) while pumping protons (H^+^) across the inner mitochondrial membrane to establish a proton gradient. Natural products target complex III by interfering with UQH_2_ binding to *cytochrome bc_1_*, blocking electron transfer and proton pumping. This disruption collapses the mitochondrial membrane potential, impairs ATP generation, and ultimately inhibits *Babesia* energy metabolism, leading to parasite death. **(B)** Schematic diagram of *Babesia* lactate dehydrogenase (LDH) inhibition by natural products and derivatives. In the catalytic process, LDH utilizes its active pocket to bind NADH and pyruvate, catalyzing the conversion of pyruvate to lactate while oxidizing NADH to NAD^+^—a reaction essential for sustaining ATP generation in *Babesia*. Natural products such as gossypol and gossypol-like compound DHNA (3,5-dihydroxy-2-naphthoic acid) compete with NADH for binding to the active pocket of *Babesia* LDH. This competitive inhibition blocks the pyruvate-lactate conversion reaction, disrupts the parasite’s glycolytic energy metabolism, and ultimately suppresses *Babesia* proliferation and survival. **(C)** Schematic diagram of oxidative stress induction in *Babesia* by natural products via the Fenton reaction. *Babesia* degrades hemoglobin during erythrocytic proliferation, releasing iron ions (Fe³^+^) and protoporphyrin IX (PPIX, forming Fe(III)-PPIX complexes). Natural products (e.g., quinoline derivatives, artemisinin derivatives) promote the reduction of Fe³^+^ to Fe²^+^. Fe²^+^ then participates in the Fenton reaction with hydrogen ions (H^+^) and reactive oxygen species (ROS) precursors, generating highly toxic hydroxyl radicals (OH-). These OH- radicals attack the labile hydrogen in unsaturated fatty acids of *Babesia* membrane phospholipids, inducing lipid peroxidation.

The energy metabolism of *Babesia* parasites is central to their survival and replication, making key metabolic enzymes attractive targets for antiparasitic drug development (Gomez-Gonzalez et al., 2024). Research has particularly focused on the mitochondrial electron transport chain and glycolysis (Shikano et al., 1998; Li et al., 2024). Mitochondrial complex III is critical for electron transfer and ATP production, catalyzing electrons from reduced ubiquinone to *cytochrome c* while generating a proton gradient across the inner mitochondrial membrane. Inhibiting complex III disrupts this process, collapsing the mitochondrial membrane potential and impairing energy metabolism, ultimately killing the parasite (Komatsuya et al., 2022; Sheokand et al., 2024). In prior studies, chalcone has been shown to interfere with the binding of ubiquinone to *cytochrome bc_1_*, thereby attenuating the associated oxidation-reduction reactions. Additionally, this compound inhibits the enzymatic activity of fumarate reductase, which disrupts the energy metabolic pathways essential for parasite survival and ultimately results in parasite death. Trans-chalcone is hypothesized to exert a comparable mode of action to chalcone, owing to the structural similarity shared between these two compounds (Batiha et al., 2019). The detailed molecular mechanism underlying the inhibition of mitochondrial complex III and its subsequent effects on *Babesia* energy metabolism is illustrated in **Figure 2A**.

*Babesia* lacks a conventional tricarboxylic acid cycle, relying mainly on anaerobic glycolysis. LDH catalyzes the conversion of pyruvate to lactate, playing a key role in ATP production under these conditions (Choi et al., 2024; Luo et al., 2025). Gossypol and its derivatives inhibit *Babesia* LDH by competing with NADH at the active site, preventing pyruvate-lactate conversion (**Figure 2B**). Crystallographic studies of *B. orientalis* LDH revealed a unique catalytic pocket sensitive to gossypol-like compounds, which differs from mammalian LDH and offers a selective target for drug design (Yu et al., 2022; Luo et al., 2025). Targeting mitochondrial function and glycolytic enzymes represents a promising strategy for selective inhibition of *Babesia* energy metabolism, forming the basis for both current and next-generation antiparasitic therapies.

### 4.2 Induction of oxidative stress

*Babesia*’s metabolic activity during the erythrocytic stage involves hemoglobin degradation and oxidative stress responses. *Babesia* degrades hemoglobin to release iron ions, generating substantial ROS that activate the host’s oxidative defense system (Srinivasan et al., 2021). Unlike red blood cells, which possess robust antioxidant systems, *Babesia* lacks glutathione redox cycle and relies solely on the thioredoxin system, rendering the parasite more vulnerable to oxidative damage (**Figure 2C**). Metal-chelating quinoline derivatives, such as ferroquine, exploit this vulnerability. Their active divalent iron center engages in redox reactions with free or loosely bound iron ions near or within the parasite, catalyzing hydroxyl radical formation via the Fenton reaction. These radicals attack unsaturated fatty acids in membrane phospholipids, triggering lipid peroxidation, membrane disruption, increased permeability, and ultimately parasite death (Jones et al., 2015). Fluorescence labeling studies have shown that quinoline compounds, including chloroquine, interact with both parasite membrane lipids and multidrug resistance-associated proteins, disrupting ion homeostasis and membrane integrity, a mechanism likely linked to oxidative stress (Woodland et al., 2018). 8-Aminoquinoline compounds, such as tafenoquine, induce ROS accumulation in *Babesia*, triggering upregulation of Trx-cycle antioxidant enzymes. Elevated ROS levels subsequently damage parasite membranes, nucleic acids, and organelles, producing vacuole-like abnormal phenotypes similar to hydrogen peroxide treatment (Huang et al., 2018). Other apicomplexan-targeting agents, such as primaquine and sitamaquine, exert antiparasitic effects via oxidative damage: in *P. falciparum*, CYP2D6-mediated metabolism of primaquine generates hydroxyl metabolites that oxidize to quinonemines, producing H_2_O_2_. Quinoneimines serve as substrates for cytochrome P_450_ NADPH: oxidoreductase reduction, further amplifying ROS formation, which damages protein sulfhydryl groups and Fe/FeS-containing enzymes (Camarda et al., 2019).

Artemisinin derivatives, including artesunate, primarily act by generating free radicals that disrupt the redox balance of parasites. *In vivo* studies indicate that artesunate can enhance host hepatic antioxidant enzyme activity and reduce lipid peroxidation, either as a host adaptive response or due to decreased oxidative stress following parasite clearance. Critically, *Babesia*’s limited antioxidant defenses—such as the absence of the pentose phosphate pathway—render it highly susceptible to oxidative stress induced by artemisinin, forming the basis for its selective antiparasitic activity (Iguchi et al., 2015; Zheng et al., 2025).

## 5 Advantages and challenges of natural products against babesiosis control

### 5.1 Broad-spectrum activity and application advantages

A key advantage of natural products lies in their structural diversity and multi-mechanistic activity, which may partially address the major limitations of conventional therapies. Chalcone derivatives reportedly bind mitochondrial complex III to disrupt ATP generation, and they exhibit a high therapeutic, catechin hydrate appears to combine “metabolic suppression” and “oxidative stress induction” while potentially reducing selection for resistance-conferring mutations (Batiha et al., 2019; Camarda et al., 2020), index that supports their promise as lead scaffolds. In addition, several natural products exhibit host-beneficial properties beyond parasite inhibition. Baicalein may mitigate infection-associated hepatic inflammation, while ellagic acid may alleviate hemolysis, suggesting a host-protective property that is less typical of standard chemotherapeutics (Chen et al., 2024a; Chen et al., 2024b).

Nevertheless, it is critical to recognize that many favorable signals originate from standardized *in vitro* systems that differ substantially from the complexity of *in vivo* microenvironments. *In vitro* assays typically involve high erythrocyte purity and absence of immune components, whereas *in vivo* efficacy is shaped by host immune status, hemodynamics, and tissue barriers, all of which can modulate drug exposure and parasite clearance (Brown and Guler, 2020). For example, baicalein exhibits potent *in vitro* inhibitory activity against *B. duncani*; however, *in vitro* subculture assays demonstrate that it merely suppresses parasitic proliferation without achieving complete eradication, even at a high concentration of 8×IC_50_. In the same study, artemisinin-class compounds may display moderate *in vitro* inhibition but limited *in vivo* killing, potentially because *Babesia* lacks the heme metabolism features characteristic of *Plasmodium* (Zhang et al., 2021). Collectively, these gaps underscore the need for more clinically relevant experimental systems to support robust evaluation of natural product candidates.

### 5.2 Challenges and translational barriers in clinical application

The limitations of monotherapy for babesiosis remain evident. From a pragmatic clinical perspective, natural products are more plausibly positioned as combination potentiators rather than stand-alone therapeutic replacements. Numerous highly active compounds lack the capacity to achieve complete *in vivo Babesia* eradication. Catechin hydrate can suppress *B. gibsoni* proliferation in canine models, yet low-level parasitemia may remain detectable on blood smears, failing to achieve a sterilizing cure (Batiha et al., 2020). Similarly, Artesunate shows good *in vitro* activity against *B. gibsoni*, but relapse occurs frequently when administered as a monotherapy, often necessitating combination regimens and extended treatment courses (Loo et al., 2017). Consequently, natural products are unlikely to replace established combination therapies in the near term; their principal value resides in optimizing current strategies by reducing required doses, minimizing adverse effects, and delaying the emergence of drug resistance (El-Readi et al., 2021; Wu et al., 2023; Kincses et al., 2024).

The failure of highly active *in vitro* candidates to translate effectively *in vivo* typically stems from inadequate intraerythrocytic exposure, rapid metabolic elimination, and host toxicity or drug-drug interactions. These factors collectively represent the primary barriers to clinical translation (Siqueira-Neto et al., 2023). Numerous natural products are hampered by poor stability and low bioavailability, which precludes their ability to attain optimal IC_50_ values *in vivo*. For example, oral administration of ellagic acid results in cleavage of its lactone ring by intestinal microbiota, leading to rapid metabolism, a short plasma half-life, and consequently suboptimal inhibitory efficacy. The development of sustained-release nanoformulations emerges as a pivotal strategy to surmount these translational barriers (Rodrigues MP. et al., 2023).

Mechanistic ambiguity constitutes another critical bottleneck for natural products, restricting their rational optimization and targeted design. For numerous high-potency molecules, definitive molecular targets remain elusive. Cephaeline hydrobromide, emetine hydrochloride, harringtonine, and lycorine, among other natural products, exhibit IC_50_ values of less than 1 μM against *B. gibsoni* (Ji et al., 2021). However, no consensus has been reached within the academic community regarding their primary mechanism of action to date. Furthermore, while multi-mechanistic activity may mitigate the risk of parasite resistance, it complicates the prediction of off-target effects and associated liabilities. For instance, gossypol potently inhibits parasitic LDH yet is associated with reproductive toxicity in mammals, underscoring the need for careful balancing of retained anti-parasitic potency and reduced host toxicity during the optimization of derivatives (Gao et al., 2022; Yu et al., 2024).

A lack of standardization and challenges in quality control further impede the translational progress of natural products targeting *Babesia* (Sorkin et al., 2020). To date, no unified framework exists for *in vitro* anti-*Babesia* efficacy evaluation. Readout time points for IC_50_ vary widely, infection rates and incubation conditions are inconsistent across studies, and cross-study comparisons are therefore rendered unreliable. Additionally, the purity of natural product preparations can differ substantially. The quality control of commercially available natural product extracts is inconsistent, and impurities are highly likely to compromise both their efficacy and safety profiles. *In vivo* setting, heterogeneity also characterizes animal model selection (Noviana et al., 2022). Recent studies, however, have begun to move the field toward greater standardization. These include the development of an alternative culture medium for continuous *Babesia duncani* propagation in human erythrocytes (Vydyam and Ben Mamoun, 2026b) and a standardized culture medium and workflow for comparative drug efficacy evaluation across *Plasmodium* and *Babesia* species, which is compatible with fluorescence-based high-throughput screening (Singh et al., 2022). Thus, these advances provide a practical foundation for more reproducible anti-*Babesia* drug testing and may accelerate the translational evaluation of natural product-derived candidates.

### 5.3 Future perspectives

High-throughput screening, AI-enabled computational approaches, and advanced models should be regarded not as fully mature solutions, but as a potential toolbox for addressing the efficiency and precision challenges inherent in natural product discovery against babesiosis. Their promise is substantial, yet their current impact remains restricted by limitations in data quality, model standardization, and implementation costs. High-throughput screening (HTS) platforms based on luciferase reporters and flow cytometry may help overcome the low throughput of conventional *in vitro* evaluation methods. Luciferase-tagged *B. divergens* strains allow 96-well assays to test thousands of compounds simultaneously, reducing screening timelines from months to weeks (Rizk et al., 2016). Integration with microfluidic blood assay platforms can further enhance physiological relevance by more accurately capturing erythrocyte penetration and intracellular parasite killing, thereby reducing false-positive and false-negative results commonly observed in traditional two-dimensional assays (Li et al., 2025). Nonetheless, HTS infrastructure is expensive to establish, and large-scale screening is frequently limited by the availability and purity of natural products. This constraint is especially evident for molecules derived from rare raw materials or those with complex chemical structures, such as naphthylisoquinoline alkaloids (Shang et al., 2025). As a result, the application of HTS in babesiosis research is still at an early stage.

AI and computational biology can facilitate target prediction and structural optimization of natural products. Based on available structures of key metabolic enzymes molecular docking and model-guided design can help infer binding modes and direct structural modifications to improve ligand affinity. For example, AI-guided optimization of DHNA derivatives has been reported to increase binding affinity to *B. microti* LDH by approximately two-fold relative to the parent compound (Luo et al., 2025). Deep learning architectures such as convolutional neural networks and Transformers may also prioritize candidate molecules from large natural product libraries by leveraging known activity patterns, thereby reducing empirical trial-and-error. Importantly, virtual screening hits still require extensive experimental validation, and robust prediction validation closed-loop workflows remain underdeveloped (Yuan et al., 2023; Jiang et al., 2025).

Furthermore, AI and computational biology technologies should be fully integrated into research workflows, deep learning approaches can applied to virtual screening, target prediction, and structural optimization of natural product derivatives, while machine learning-guided phenotypic screening can facilitate high-throughput identification of anti-*Babesia* agents (Jia et al., 2022). The development of synergistic combination therapies involving natural products and existing chemotherapeutic drugs should be prioritized. By leveraging pathway complementarity among different agents, highly effective and low-toxicity combination regimens should be identified to address the clinical challenges of drug resistance and low radical cure rates associated with monotherapy (Chen and Hsiang, 2022). Additionally, personalized treatment strategies tailored to diverse host populations, including immunocompromised individuals and livestock, should be explored to mitigate the escalating prevention and control challenges posed by the global spread of babesiosis via vector transmission.

## 6 Conclusions

Natural products and their derivatives, characterized by structural diversity and multi-mechanistic synergy, represent an important resource for addressing key limitations of current babesiosis therapy, including drug resistance, narrow activity spectra, and dose-limiting toxicity. Alkaloids, sesquiterpenes, flavonoids, and polyphenols have demonstrated notable antiparasitic activity and are particularly well positioned as combination potentiators. They are capable of reducing conventional drug dosages, delaying resistance-associated mutations, and mitigating host adverse effects. Moreover, they may help limit infection-associated tissue injury, supporting potential value in high-risk populations. Nevertheless, major challenges remain: overall evidence quality is constrained by concentration on a limited number of species and *in vitro* datasets; methodological heterogeneity and insufficient quality-control standards impede cross-study synthesis; and translational barriers, especially pharmacokinetic limitations and mechanistic uncertainty, continue to restrict clinical progress.

While HTS, AI, and advanced models offer a promising enabling toolbox, their broad deployment is currently limited by data standardization and cost. Future work should prioritize on expanding foundational datasets and standardizing evaluation frameworks, clarifying mechanisms and improving pharmacokinetics through formulation and optimization, and validating synergy-driven regimens in zoonotic species and clinically relevant scenarios. With continued advances in *Babesia* genomic resources, assay harmonization, and formulation technologies, natural product-derived candidates may become an increasingly meaningful adjunct to babesiosis management, although the practical impact of AI/HTS approaches will require further empirical validation and iterative optimization to realize genuine bench-to-bedside translation.

## References

[B1] Aboge G. O. Jia H. Terkawi M. A. Goo Y. K. Nishikawa Y. Sunaga F. . (2008). Cloning, expression, and characterization of babesia gibsoni dihydrofolate reductase-thymidylate: inhibitory effect of antifolates on its catalytic activity and parasite proliferation. Antimicrob. Agents Chemother. 52, 4072–4080. doi: 10.1128/aac.00384-08 18794380 PMC2573109

[B2] AbouLaila M. Munkhjargal T. Sivakumar T. Ueno A. Nakano Y. Yokoyama M. . (2012). Apicoplast-targeting antibacterials inhibit the growth of babesia parasites. Antimicrob. Agents Chemother. 56, 3196–3206. doi: 10.1128/aac.05488-11 22391527 PMC3370714

[B3] Aderanti T. Marshall J. M. Thekkiniath J. (2025). Effect of protease inhibitors on the intraerythrocytic development of babesia microti and babesia duncani, the causative agents of human babesiosis. J. Eukaryot Microbiol. 72, e13064. doi: 10.1111/jeu.13064 39556081 PMC11780687

[B4] Akhtar N. Pradhan N. Barik G. K. Chatterjee S. Ghosh S. Saha A. . (2020). Quinine-based semisynthetic ion transporters with potential antiproliferative activities. ACS Appl. Mater. Interfaces 12, 25521–25533. doi: 10.1021/acsami.0c01259 32425038

[B5] Ali A. Zeb I. Alouffi A. Zahid H. Almutairi M. M. Ayed Alshammari F. . (2022). Host immune responses to salivary components—a critical facet of tick-host interactions. Front. Cell. Infect. Microbiol. 12, 809052. doi: 10.3389/fcimb.2022.809052 35372098 PMC8966233

[B6] Almendros A. Burchell R. Wierenga J. (2020). An alternative combination therapy with metronidazole, clindamycin and doxycycline for babesia gibsoni (asian genotype) in dogs in hong kong. J. Vet. Med. Sci. 82, 1334–1340. doi: 10.1292/jvms.20-0209 32759546 PMC7538310

[B7] Al-Nazal H. A. Cooper E. Ho M. F. Eskandari S. Majam V. Giddam A. K. . (2021). Pre-clinical evaluation of a whole-parasite vaccine to control human babesiosis. Cell Host Microbe 29, 894–903.e5. doi: 10.1016/j.chom.2021.04.008 33989514

[B8] Alzan H. F. Mahmoud M. S. Suarez C. E. (2024). Current vaccines, experimental immunization trials, and new perspectives to control selected vector borne blood parasites of veterinary importance. Front. Vet. Sci. 11, 1484787. doi: 10.3389/fvets.2024.1484787 39606652 PMC11602000

[B9] Asquith M. Prior S. Brüning-Richardson A. (2025). Human babesiosis: the past, present and future. Expert Rev. Mol. Med. 27, e30. doi: 10.1017/erm.2025.10016 40908571 PMC12558630

[B10] Barnes G. L. Magann N. L. Perrotta D. Hörmann F. M. Fernandez S. Vydyam P. . (2024). A divergent synthesis of numerous pyrroloiminoquinone alkaloids identifies promising antiprotozoal agents. J. Am. Chem. Soc 146, 29883–29894. doi: 10.1021/jacs.4c11897 39412402 PMC11528414

[B11] Bastos R. G. Capelli-Peixoto J. Laughery J. M. Suarez C. E. Ueti M. W. (2023). Vaccination with an *in vitro* culture attenuated babesia bovis strain safely protects highly susceptible adult cattle against acute bovine babesiosis. Front. Immunol. 14, 1219913. doi: 10.3389/fimmu.2023.1219913 37583702 PMC10424928

[B12] Batiha G.-S. Beshbishy A. M. Alkazmi L. M. Nadwa E. H. Rashwan E. K. Yokoyama N. . (2020). *In vitro* and *in vivo* growth inhibitory activities of cryptolepine hydrate against several babesia species and theileria equi. PloS Negl.Trop. Dis. 14, e0008489. 32853247 10.1371/journal.pntd.0008489 PMC7451656

[B13] Batiha G.-S. Beshbishy A. M. Tayebwa D. S. Adeyemi O. S. Shaheen H. Yokoyama N. . (2019). The effects of trans-chalcone and chalcone 4 hydrate on the growth of babesia and theileria. PloS Negl.Trop. Dis. 13, e0007030. doi: 10.71086/iajh/v12i4/iajh1216 31125333 PMC6534319

[B14] Begley D. W. Edwards T. E. Raymond A. C. Smith E. R. Hartley R. C. Abendroth J. . (2011). Inhibitor-bound complexes of dihydrofolate reductase-thymidylate synthase from Babesia bovis. Acta Crystallogr. Sect. F Struct. Biol. Commun. 67, 1070–1077. doi: 10.1107/s1744309111029009 21904052 PMC3169404

[B15] Beshbishy A. M. Batiha G.-S. Yokoyama N. Igarashi I. (2019). Ellagic acid microspheres restrict the growth of babesia and theileria *in vitro* and babesia microti *in vivo*. Parasit Vectors 12, 269. doi: 10.1186/s13071-019-3520-x 31138282 PMC6537213

[B16] Birkholtz L. M. Alano P. Leroy D. (2022). Transmission-blocking drugs for malaria elimination. Trends Parasitol. 38, 390–403. doi: 10.1016/j.pt.2022.01.011 35190283

[B17] Bonnet P. L. Hoffmann C. V. Le Nan N. Bellamy L. Hoarau G. Flori P. . (2023). Atovaquone exposure and pneumocystis jirovecii cytochrome b mutations: French data and review of the literature. Med. Mycol. 61, myad095. doi: 10.1093/mmy/myad095 37656874

[B18] Bringmann G. Fayez S. Shamburger W. Feineis D. Winiarczyk S. Janecki R. . (2020). Naphthylisoquinoline alkaloids and their synthetic analogs as potent novel inhibitors against babesia canis *in vitro*. Vet. Parasitol. 283, 109177. doi: 10.1016/j.vetpar.2020.109177 32629205

[B19] Brown A. C. Guler J. L. (2020). From circulation to cultivation: plasmodium *in vivo* versus *in vitro*. Trends Parasitol. 36, 914–926. doi: 10.1016/j.pt.2020.08.008 32958385

[B20] Bueno J. M. Herreros E. Angulo-Barturen I. Ferrer S. Fiandor J. M. Gamo F. J. . (2012). Exploration of 4(1h)-pyridones as a novel family of potent antimalarial inhibitors of the plasmodial cytochrome bc_1_. Future Med. Chem. 4, 2311–2323. doi: 10.4155/fmc.12.177 23234553

[B21] Callow L. L. Mellors L. T. (1966). A new vaccine for babesia Argentina infection prepared in splenectomised calves. Aust. Vet. J. 42, 464–465. doi: 10.1111/j.1751-0813.1966.tb14476.x 5962488

[B22] Camarda G. Jirawatcharadech P. Priestley R. S. Saif A. March S. Wong M. H. L. . (2019). Antimalarial activity of primaquine operates via a two-step biochemical relay. Nat. Commun. 10, 3226. doi: 10.1038/s41467-019-11239-0 31324806 PMC6642103

[B23] Camarda G. Jirawatcharadech P. Priestley R. S. Saif A. March S. Wong M. H. L. . (2020). Antibacterial green tea catechins from a molecular perspective: mechanisms of action and structure-activity relationships. Food Funct. 11, 9370–9396. doi: 10.1371/journal.pntd.0008489 33094767

[B24] Capasso C. Supuran C. T. (2023). The management of babesia, amoeba and other zoonotic diseases provoked by protozoa. Expert Opin. Ther. Pat. 33. doi: 10.3389/fped.2022.1039153 37078205

[B25] Checa R. Montoya A. Ortega N. González-Fraga J. L. Bartolomé A. Gálvez R. . (2017). Efficacy, safety and tolerance of imidocarb dipropionate versus atovaquone or buparvaquone plus azithromycin used to treat sick dogs naturally infected with the babesia microti-like piroplasm. Parasit Vectors 10, 145. doi: 10.1186/s13071-017-2049-0 28292316 PMC5404670

[B26] Chen I. Hsiang M. S. (2022). Triple artemisinin-based combination therapies for malaria: a timely solution to counter antimalarial drug resistance. Lancet Infect. Dis. 22, 751–753. doi: 10.1016/s1473-3099(21)00748-9 35276063

[B27] Chen J. Zhang Q. Xu W. Li Z. Chen X. Luo Q. . (2024a). Baicalein upregulates macrophage trem2 expression via trkb-creb1 pathway to attenuate acute inflammatory injury in acute-on-chronic liver failure. Int. Immunopharmacol. 139, 112685. doi: 10.1016/j.intimp.2024.112685 39047449

[B28] Chen J. Zhang Q. Xu W. Li Z. Chen X. Luo Q. . (2024b). Baicalein upregulates macrophage trem2 expression via trkb-creb1 pathway to attenuate acute inflammatory injury in acute-on-chronic liver failure. Int. Immunopharmacol. 139, 112685. doi: 10.1016/j.intimp.2024.112685 39047449

[B29] Choi J. W. Choi M. J. Kim Y. J. Kim S. Y. (2024). Cloning, expression, purification, and characterization of lactate dehydrogenase from plasmodium knowlesi: a zoonotic malaria parasite. Int. J. Mol. Sci. 25, 5615. doi: 10.3390/ijms25115615 38891805 PMC11171812

[B30] Choi H. I. Ko H. Y. Shin I. S. Kim H. J. (2019). Malarone® induced pancreatitis and alopecia in a dog: a case report. BMC Vet. Res. 15, 314. doi: 10.1186/s12917-019-2056-9 31477120 PMC6720934

[B31] Davis R. A. Buchanan M. S. Duffy S. Avery V. M. Charman S. A. Charman W. N. . (2012). Antimalarial activity of pyrroloiminoquinones from the Australian marine sponge zyzzya sp. J. Med. Chem. 55, 586–5858. doi: 10.1021/jm3002795 22686608

[B32] Dong Y. Liu L. Han J. Zhang L. Wang Y. Li J. . (2022). Worldwide research trends on artemisinin: a bibliometric analysis from 2000 to 2021. Front. Med. 9, 868087. doi: 10.3389/fmed.2022.868087 35602470 PMC9121127

[B33] Dunay I. R. Gajurel K. Dhakal R. Liesenfeld O. Montoya J. G. (2018). Treatment of toxoplasmosis: historical perspective, animal models, and current clinical practice. Clin. Microbiol. Rev. 31 (4), e00057–17. doi: 10.1128/cmr.00057-17 30209035 PMC6148195

[B34] Dziekan J. M. Yu H. Chen D. Dai L. Wirjanata G. Larsson A. . (2019). Identifying purine nucleoside phosphorylase as the target of quinine using cellular thermal shift assay. Sci. Transl. Med. 11, eaau3174. doi: 10.1126/scitranslmed.aau3174 30602534

[B35] El-Readi M. Z. Al-Abd A. M. Althubiti M. A. Almaimani R. A. Al-Amoodi H. S. Ashour M. L. . (2021). Multiple molecular mechanisms to overcome multidrug resistance in cancer by natural secondary metabolites. Front. Pharmacol. 12, 658513. doi: 10.3389/fphar.2021.658513 34093189 PMC8176113

[B36] Fazilani S. A. An W. Li S. Hassan M. F. Ishfaq M. Lakho S. A. . (2024). Unrevealing the therapeutic potential of artesunate against emerging zoonotic babesia microti infection in the murine model. Front. Vet. Sci. 11, 1383291. doi: 10.3389/fvets.2024.1383291 38784653 PMC11111996

[B37] Feng Y. Li Y. Zhou D. Li B. Chen G. Li N. (2022). Glycyrrhetinic acid reverses antibiotic-induced intestinal epithelial injury through RNA-binding protein human antigen R (HuR). Phytomedicine 94, 153836. doi: 10.1016/j.phymed.2021.153836 34775357

[B38] Ferrer P. Tripathi A. K. Clark M. A. Hand C. C. Rienhoff H. Y. Sullivan D. J. (2012). Antimalarial iron chelator, fbs0701, shows asexual and gametocyte plasmodium falciparum activity and single oral dose cure in a murine malaria model. PloS One 7, e37171. doi: 10.1371/journal.pone.0037171 22629364 PMC3357340

[B39] Firth A. Prathapan P. (2020). Azithromycin: the first broad-spectrum therapeutic. Eur. J. Med. Chem. 207, 112739. doi: 10.1016/j.ejmech.2020.112739 32871342 PMC7434625

[B40] Forkuo A. D. Ansah C. Boadu K. M. Boampong J. N. Ameyaw E. O. Gyan B. A. . (2016). Synergistic anti-malarial action of cryptolepine and artemisinins. Malar. J. 15, 89. doi: 10.1186/s12936-016-1137-5 26879905 PMC4754817

[B41] Freyburger L. Lemaitre L. Médaille C. Oberli F. Fanchon L. Bergamo P. (2011). Étude comparative de l’innocuité de deux vaccins commerciaux contre la babésiose canine provoquée par babesia canis. Parasite 18, 311–318. doi: 10.1039/d0fo02054k 22091461 PMC4897733

[B42] Fu B. K. Tang T. Yue M. Chen J. J. Su H. Huang X. B. . (2025). Mapping the global distribution of Babesia infections. Transbound Emerg. Dis. 2025, 5889219. doi: 10.1155/tbed/5889219 41333613 PMC12668841

[B43] Fuller L. (2024). Continuous *in vitro* propagation of babesia microti. Infect. Immun. 92, e00481-23. doi: 10.2140/pjm.1968.25.495 38837339 PMC11238550

[B44] Galappaththy G. N. Tharyan P. Kirubakaran R. (2013). Primaquine for preventing relapse in people with plasmodium vivax malaria treated with chloroquine. Cochrane Database Syst. Rev. doi: 10.1002/14651858.cd004389.pub3 24163057 PMC6532739

[B45] Gao Y. Tai W. Wang X. Jiang S. Debnath A. K. Du L. . (2022). A gossypol derivative effectively protects against zika and dengue virus infection without toxicity. BMC Biol. 20, 143. doi: 10.1186/s12915-022-01344-w 35706035 PMC9202104

[B46] Ghosh A. Bhattacharya T. Mandal D. Dutta K. Dey S. Saha K. . (2025). Synthesis of yttria nanoparticle-loaded electrospun nanofibers for enhanced antimicrobial activity, biofilm inhibition, and alleviation of diabetic wounds. ACS Appl. Bio Mater. 8, 2287–2298. doi: 10.1021/acsabm.4c01818 40009776

[B47] Gimenez A. M. Françoso K. S. Ersching J. Icimoto M. Y. Oliveira V. Rodriguez A. E. . (2016). A recombinant multi-antigen vaccine formulation containing babesia bovis merozoite surface antigens msa-2a1, msa-2b and msa-2c elicits invasion-inhibitory antibodies and ifn-γ producing cells. Parasit Vectors 9, 577. doi: 10.1186/s13071-016-1862-1 27842609 PMC5109680

[B48] Gomez-Gonzalez P. J. Gupta A. Drought L. G. Patel A. Okombo J. Van Der Watt M. . (2024). Inhibitors of malaria parasite cyclic nucleotide phosphodiesterases block asexual blood-stage development and mosquito transmission. Sci. Adv. 10, eadq1383. doi: 10.1126/sciadv.adq1383 39642214 PMC11623267

[B49] He L. Bastos R. G. Yu L. Laughery J. M. Suarez C. E. (2022). Lactate dehydrogenase as a potential therapeutic drug target to control babesia bigemina. Front. Cell. Infect. Microbiol. 12, 870852. doi: 10.3389/fcimb.2022.870852 35521220 PMC9062099

[B50] Hiben M. G. Sibhat G. G. Fanta B. S. Gebrezgi H. D. Tesema S. B. (2015). Evaluation of senna singueana leaf extract as an alternative or adjuvant therapy for malaria. J. Tradit. Complement Med. 6, 112–117. doi: 10.1016/j.jtcme.2014.11.014 26870688 PMC4737944

[B51] Holbrook N. R. Klontz E. H. Adams G. C. Schnittman S. R. Issa N. C. Bond S. A. . (2023). Babesia microti variant with multiple resistance mutations detected in an immunocompromised patient receiving atovaquone prophylaxis. Open Forum Infect. Dis. 10, ofad097. doi: 10.1051/parasite/2011184311 36968958 PMC10034591

[B52] Huang L. Sun Y. Huo D. D. Xu M. Xia L. Y. Yang N. . (2023). Successful treatment with doxycycline monotherapy for human infection with babesia venatorum (babesiidae, sporozoa) in China: a case report and proposal for a clinical regimen. Infect. Dis. Poverty 12, 67. doi: 10.1186/s40249-023-01111-1 37443058 PMC10339522

[B53] Huang J. Xiong K. Zhang H. Zhao Y. Cao J. Gong H. . (2018). Molecular characterization of babesia microti thioredoxin (bmtrx2) and its expression patterns induced by antiprotozoal drugs. Parasit Vectors 11, 38. doi: 10.2139/ssrn.5290723 29335000 PMC5769273

[B54] Iguchi A. Matsuu A. Fujii Y. Ikadai H. Hikasa Y. (2013). The *in vitro* interactions and *in vivo* efficacy of atovaquone and proguanil against Babesia gibsoni infection in dogs. Vet. Parasitol. 197, 527–533. doi: 10.1093/ofid/ofad097 24075418

[B55] Iguchi A. Matsuu A. Matsuyama K. Hikasa Y. (2015). The efficacy of artemisinin, artemether, and lumefantrine against babesia gibsoni *in vitro*. Parasitol. Int. 64, 190–193. doi: 10.1016/j.parint.2014.12.006 25523292

[B56] Jerzak M. Gandurski A. Tokaj M. Stachera W. Szuba M. Dybicz M. . (2023). Advances in babesia vaccine development: an overview. Pathogens 12, 300. doi: 10.3390/pathogens12020300 36839572 PMC9962624

[B57] Jhulki S. Bhowmik B. Pal A. (2025). Enlightening the promising role of nanoparticle-based treatments against naegleria fowleri-induced primary amoebic meningoencephalitis: a brain-eating disease. Microb. Pathog. 199, 107234. doi: 10.1016/j.micpath.2024.107234 39701479

[B58] Ji S. Liu M. Galon E. M. Rizk M. A. Li J. Li Y. . (2021). *In vitro* screening of novel anti-babesia gibsoni drugs from natural products. Parasitol. Int. 85, 102437. doi: 10.1016/j.vetpar.2013.06.006 34389492

[B59] Jia X. N. Wang W. J. Yin B. Zhou L. J. Zhen Y. Q. Zhang L. . (2022). Deep learning promotes the screening of natural products with potential microtubule inhibition activity. ACS Omega 7, 28334–28354. doi: 10.1021/acsomega.2c02854 35990425 PMC9386835

[B60] Jiang J. Chen L. Ke L. Dou B. Zhang C. Feng H. . (2025). A review of transformer models in drug discovery and beyond. J. Pharm. Anal. 15, 101081. doi: 10.1016/j.jpha.2024.101081 40635975 PMC12240084

[B61] Jones R. A. Panda S. S. Hall C. D. (2015). Quinine conjugates and quinine analogues as potential antimalarial agents. Eur. J. Med. Chem. 97, 335–355. doi: 10.1016/j.ejmech.2015.02.002 25683799

[B62] Kincses A. Ghazal T. S. A. Hohmann J. (2024). Synergistic effect of phenylpropanoids and flavonoids with antibiotics against Gram-positive and Gram-negative bacterial strains. Pharm. Biol. 62, 659–665. doi: 10.1080/13880209.2024.2389105 39126171 PMC11318484

[B63] Kirk S. K. Levy J. K. Crawford P. C. (2017). Efficacy of azithromycin and compounded atovaquone for treatment of babesia gibsoni in dogs. J. Vet. Intern. Med. 31, 1108–1112. doi: 10.1111/jvim.14777 28625019 PMC5508350

[B64] Kletsova E. A. Spitzer E. D. Fries B. C. Marcos L. A. (2017). Babesiosis in long island: review of 62 cases focusing on treatment with azithromycin and atovaquone. Ann. Clin. Microbiol. Antimicrob. 16, 26. doi: 10.1186/s12941-017-0198-9 28399851 PMC5387270

[B65] Komatsuya K. Sakura T. Shiomi K. Ōmura S. Hikosaka K. Nozaki T. . (2022). Siccanin is a dual-target inhibitor of plasmodium falciparum mitochondrial complex II and complex III. Pharmaceuticals 15, 903. doi: 10.3390/ph15070903 35890202 PMC9319939

[B66] Koonyosying P. Srichairatanakool S. Tiwananthagorn S. Sthitmatee N. (2023). Inhibitory effects on bovine babesial infection by iron chelator, 1-(n-acetyl-6-aminohexyl)-3-hydroxy-2-methylpyridin-4-one (cm1), and antimalarial drugs. Vet. Parasitol. 324, 110055. doi: 10.1016/j.vetpar.2023.110055 37931475

[B67] Krause P. J. Rogers R. Shah M. K. Kang H. Parsonnet J. Kodama R. . (2024). Tafenoquine for relapsing babesiosis: a case series. Clin. Infect. Dis. 79, 130–137. doi: 10.1093/cid/ciae238 38814096 PMC11259219

[B68] Kumar A. O’Bryan J. Krause P. J. (2021). The global emergence of human babesiosis. Pathogens 10, 1447. doi: 10.3390/pathogens10111447 34832603 PMC8623124

[B69] Lawres L. A. Garg A. Kumar V. Bruzual I. Forquer I. P. Renard I. . (2016). Radical cure of experimental babesiosis in immunodeficient mice using a combination of an endochin-like quinolone and atovaquone. J. Exp. Med. 213, 1307–1318. doi: 10.1084/jem.20151519 27270894 PMC4925016

[B70] Lemieux J. E. Tran A. D. Freimark L. Schaffner S. F. Goethert H. Andersen K. G. . (2016). A global map of genetic diversity in babesia microti reveals strong population structure and identifies variants associated with clinical relapse. Nat. Microbiol. 1, 16079. doi: 10.1038/nmicrobiol.2016.79 27572973 PMC5563076

[B71] Li C. Bache E. G. Apgar A. L. Tufts D. M. Niepa T. H. R. (2025). *In vitro* monitoring of babesia microti infection dynamics in whole blood microenvironments. Adv. Sci. 12, e08185. doi: 10.1016/j.parint.2021.102437 PMC12591138 40788132

[B72] Li D. F. Wang L. N. Bai Y. D. Yu Y. X. Lu X. Guan X. A. . (2024). Phosphoenolpyruvate carboxylase (pepc) is essential for the glycolytic pathway and parasite proliferation in babesia gibsoni. Anim. Dis. 4, 44. doi: 10.1186/s44149-024-00148-5 38164791

[B73] Li X. Zhang J. Lin S. Xing Y. Zhang X. Ye M. . (2022). (+)-catechin, epicatechin and epigallocatechin gallate are important inducible defensive compounds against ectropis grisescens in tea plants. Plant Cell Environ. 45, 496–511. doi: 10.1111/pce.14216 34719788

[B74] Li W. J. Zhang L. Wu H. X. Li M. Wang T. Zhang W. B. . (2022). Intestinal microbiota mediates gossypol-induced intestinal inflammation, oxidative stress, and apoptosis in fish. J. Agric. Food. Chem. 70, 6688–6697. doi: 10.1021/acs.jafc.2c01263 35635005

[B75] Liang R. Rao H. Pang Q. Xu R. Jiao Z. Lin L. . (2023). Human apoe2 protects mice against plasmodium berghei anka experimental cerebral malaria. MBio 14, e02346-23. doi: 10.1128/mbio.02346-23 37874152 PMC10746236

[B76] Liu M. Ji S. Kondoh D. Galon E. M. Li J. Tomihari M. . (2021). Tafenoquine is a promising drug candidate for the treatment of babesiosis. Antimicrob. Agents Chemother. 65, e00204-21. doi: 10.1128/aac.00204-21 33941516 PMC8373227

[B77] Liu P. C. Lin Y. L. Lin C. N. Su B. L. (2016). A SimpleProbe(®) real-time PCR assay for differentiating the cytochrome b M121I mutation in clinical specimens from dogs infected with Babesia gibsoni. Ticks Tick-borne Dis. 7, 639–643. doi: 10.1016/j.ttbdis.2016.02.009 26874668

[B78] Loo C. S. N. Lam N. S. K. Yu D. Su X. Lu F. (2017). Artemisinin and its derivatives in treating protozoan infections beyond malaria. Pharmacol. Res. 117, 192–217. doi: 10.1016/j.phrs.2016.11.012 27867026 PMC5316320

[B79] Lu Y. Hu Z. Shao F. Song M. Hang T. (2021). Simultaneous determination of tazarotene, clindamycin phosphate and their active metabolites in Bama mini-pig skin by LC-MS/MS: application to the development of a tazarotene/clindamycin phosphate cream. J. Chromatogr. B 1162, 122455. doi: 10.1016/j.jchromb.2020.122455 33360677

[B80] Luo W. Yu L. Lu S. Yu Y. Bai Y. Wang S. . (2025). Design and screening of novel molecular compounds targeting lactate dehydrogenase of babesia microti. Parasit Vectors 18, 69. doi: 10.1186/s13071-024-06623-9 39988659 PMC11847361

[B81] Ma R. Yue C. Gu J. Wu W. Hou R. Huang W. . (2024). Efficacy of azithromycin combined with compounded atovaquone in treating babesiosis in giant pandas. Parasit Vectors 17, 531. doi: 10.1186/s13071-024-06615-9 39716228 PMC11665231

[B82] Ma N. Zhang Z. Liao F. Jiang T. Tu Y. (2020). The birth of artemisinin. Pharmacol. Ther. 216, 107658. doi: 10.1016/j.pharmthera.2020.107658 32777330

[B83] Mahoney D. F. (1967). Bovine babesiosis: the immunization of cattle with killed babesia Argentina. Exp. Parasitol. 20, 125–129. doi: 10.1016/0014-4894(67)90030-6 4961048

[B84] Marcos L. A. Wormser G. P. (2023). Relapsing babesiosis with molecular evidence of resistance to certain antimicrobials commonly used to treat babesia microti infections. Open Forum Infect. Dis. 10, ofad391. doi: 10.1093/ofid/ofad391 37539067 PMC10394720

[B85] Matsuu A. Yamasaki M. Xuan X. Ikadai H. Hikasa Y. (2008). *In vitro* evaluation of the growth inhibitory activities of 15 drugs against babesia gibsoni (aomori strain). Vet. Parasitol. 157, 1–8. doi: 10.1002/advs.202508185 18771856

[B86] Nagai A. Yokoyama N. Matsuo T. Bork S. Hirata H. Xuan X. . (2003). Growth-inhibitory effects of artesunate, pyrimethamine, and pamaquine against Babesia equi and. Antimicrob. Agents Chemother. 47, 800–803. doi: 10.1128/aac.47.2.800-803.2003 12543697 PMC151728

[B87] Noviana E. Indrayanto G. Rohman A. (2022). Advances in fingerprint analysis for standardization and quality control of herbal medicines. Front. Pharmacol. 13, 853023. doi: 10.3389/fphar.2022.853023 35721184 PMC9201489

[B88] Oliver M. E. Hinks T. S. C. (2021). Azithromycin in viral infections. Rev. Med. Virol. 31, e2163. doi: 10.1002/rmv.2163 32969125 PMC7536932

[B89] Pang J. Feng X. Liang Q. Zheng X. Duan Y. Zhang X. . (2022). Ferritin-nanocaged atp traverses the blood-testis barrier and enhances sperm motility in an asthenozoospermia model. ACS Nano 16, 4175–4185. doi: 10.1021/acsnano.1c10029 35167250

[B90] Pang H. Xue W. Shi A. Li M. Li Y. Cao G. . (2016). Multiple-ascending-dose pharmacokinetics and safety evaluation of baicalein chewable tablets in healthy chinese volunteers. Clin. Drug Investig. 36, 713–724. doi: 10.1007/s40261-016-0418-7 27352310

[B91] Qin Y. Ma S. (2020). Recent advances in the development of macrolide antibiotics as antimicrobial agents. Mini-Rev. Med. Chem. 20, 601–625. doi: 10.2174/1389557520666191223160942 31868146

[B92] Rahman S. Panicker V. P. Narayanan A. Pillai U. N. Unny M. Krishna G. . (2022). Multiple point mutations in cytochrome b gene of babesia gibsoni – a possible cause for buparvaquone resistance. Vet. Parasitol. 312, 109823. doi: 10.1016/j.vetpar.2022.109823 36306628

[B93] Renard I. Ben Mamoun C. (2021). Treatment of human babesiosis: then and now. Pathogens 10, 1120. doi: 10.3390/pathogens10091120 34578153 PMC8469882

[B94] Rizk M. A. El-Sayed S. A. AbouLaila M. Tuvshintulga B. Yokoyama N. Igarashi I. (2016). Large-scale drug screening against babesia divergens parasite using a fluorescence-based high-throughput screening assay. Vet. Parasitol. 227, 93–97. doi: 10.1016/j.vetpar.2016.07.032 27523944

[B95] Rodrigues F. F. Lino C. I. Oliveira V. L. S. Zaidan I. Melo I. S. F. Braga A. V. . (2023). A clindamycin acetylated derivative with reduced antibacterial activity inhibits articular hyperalgesia and edema by attenuating neutrophil recruitment, nf-κb activation and tumor necrosis factor-α production. Int. Immunopharmacol. 122, 110609. doi: 10.1016/j.intimp.2023.110609 37429145

[B96] Rodrigues M. P. Pinto P. N. Dias R. R. D. S. Biscoto G. L. Salvato L. A. Millán R. D. S. . (2023). The antimicrobial applications of nanoparticles in veterinary medicine: a comprehensive review. Antibiotics 12, 958. doi: 10.3390/antibiotics12060958 37370277 PMC10295505

[B97] Rodriguez J. B. Szajnman S. H. (2023). An updated review of chemical compounds with anti-toxoplasma gondii activity. Eur. J. Med. Chem. 262, 115885. doi: 10.1016/j.ejmech.2023.115885 37871407

[B98] Rogers R. Krause P. J. Norris A. M. Ting M. H. Nagami E. H. Cilley B. . (2023). Broad antimicrobial resistance in a case of relapsing babesiosis successfully treated with tafenoquine. Clin. Infect. Dis. 76, 741–744. doi: 10.1093/cid/ciac473 35684960

[B99] Sanchez E. Vannier E. Wormser G. P. Hu L. T. (2016). Diagnosis, treatment, and prevention of lyme disease, human granulocytic anaplasmosis, and babesiosis: a review. JAMA 315, 1767. doi: 10.1001/jama.2016.2884 27115378 PMC7758915

[B100] Santos J. H. M. Siddle H. V. Raza A. Stanisic D. I. Good M. F. Tabor A. E. . (2023). Exploring the landscape of babesia bovis vaccines: progress, challenges, and opportunities. Parasit Vectors 16, 274. doi: 10.18174/635504 37563668 PMC10413621

[B101] Shamsi M. Zahedi P. Ghourchian H. Minaeian S. (2017). Microfluidic-aided fabrication of nanoparticles blend based on chitosan for a transdermal multidrug delivery application. Int. J. Biol. Macromol. 99, 433–442. doi: 10.1016/j.ijbiomac.2017.03.013 28274863

[B102] Shang X. Dai L. Cao X. Ma Y. Gulnaz I. Miao X. . (2025). Natural products in antiparasitic drug discovery: advances, opportunities and challenges. Nat. Prod. Rep. 42, 1419–1458. doi: 10.1039/d5np00007f 40501439

[B103] Sheokand P. K. Pradhan S. Maclean A. E. Mühleip A. Sheiner L. (2024). Plasmodium falciparum mitochondrial complex III, the target of atovaquone, is essential for progression to the transmissible sexual stages. Int. J. Mol. Sci. 25, 9239. doi: 10.1016/j.vetpar.2008.07.023 39273187 PMC11394760

[B104] Shikano S. Katoh R. Adachi K. Bonkobara M. Inaba M. Ono K. (1998). Mitochondrial function in babesia microti and babesia rodhaini. Int. J. Parasitol. 28, 567–570. doi: 10.1016/s0020-7519(98)00009-5 9602376

[B105] Si W. Fang C. Liu C. Yin M. Xu W. Li Y. . (2023). Why is babesia not killed by artemisinin like plasmodium? Parasit Vectors 16, 193. doi: 10.64628/aam.jdcct6fy3 37291657 PMC10249562

[B106] Simon M. S. Westblade L. F. Dziedziech A. Visone J. E. Furman R. R. Jenkins S. G. . (2017). Clinical and molecular evidence of atovaquone and azithromycin resistance in relapsed babesia microti infection associated with rituximab and chronic lymphocytic leukemia. Clin. Infect. Dis. 65, 1222–1225. doi: 10.1093/cid/cix477 28541469 PMC6248624

[B107] Singh P. Lonardi S. Liang Q. Vydyam P. Khabirova E. Fang T. . (2023). Babesia duncani multi-omics identifies virulence factors and drug targets. Nat. Microbiol. 8, 845–859. 37055610 10.1038/s41564-023-01360-8 PMC10159843

[B108] Singh P. Pal A. C. Ben Mamoun C. (2022). An alternative culture medium for continuous *in vitro* propagation of the human pathogen Babesia duncani in human erythrocytes. Pathogens 11, 599. doi: 10.3390/pathogens11050599 35631120 PMC9146245

[B109] Siqueira-Neto J. L. Wicht K. J. Chibale K. Burrows J. N. Fidock D. A. Winzeler E. A. . (2023). Antimalarial drug discovery: progress and approaches. Nat. Rev. Drug Discov. 22, 807–826. doi: 10.1038/s41573-023-00772-9 37652975 PMC10543600

[B110] Sixty Degrees Pharmaceuticals (2025). 60 degrees pharma announces IRB approval of phase II study to evaluate tafenoquine for chronic babesiosis | 60 degrees pharmaceuticals, inc. Available online at: https://investors.60degreespharma.com/news-releases/news-release-details/60-degrees-pharma-announces-irb-approval-phase-ii-study-evaluate (Accessed May 24, 2025).

[B111] Smith R. P. Hunfeld K. P. Krause P. J. (2020). Management strategies for human babesiosis. Expert Rev. Anti-Infect Ther. 18, 625–636. doi: 10.1080/14787210.2020.1752193 32268823

[B112] Sorkin B. C. Kuszak A. J. Bloss G. Fukagawa N. K. Hoffman F. A. Jafari M. . (2020). Improving natural product research translation: from source to clinical trial. FASEB J. 34, 41–65. doi: 10.31232/osf.io/8vfnc 31914647 PMC7470648

[B113] Srinivasan S. Roy D. Chavas T. E. J. Vlaskin V. Ho D. K. Pottenger A. . (2021). Liver-targeted polymeric prodrugs of 8-aminoquinolines for malaria radical cure. J. Control Release 331, 213–227. doi: 10.1016/j.jconrel.2020.12.046 33378692

[B114] Tajeri S. Chattopadhyay D. Langsley G. Nijhof A. M. (2024). A theileria annulata parasite with a single mutation, methionine 128 to isoleucine (m128i), in cytochrome b is resistant to buparvaquone. PloS One 19, e0299002. doi: 10.3390/ijms25179239 38626086 PMC11020719

[B115] Tang M. Xia W. Song F. Liu C. Wang X. Zhou H. . (2024). Loss of gcn2 exacerbates gossypol induced oxidative stress, apoptosis and inflammation in zebrafish. Fish Shellfish Immunol. 151, 109727. doi: 10.1016/j.fsi.2024.109727 38936520

[B116] Tayebwa D. S. Tuvshintulga B. Guswanto A. Nugraha A. B. Batiha G.-S. Gantuya S. . (2018). The effects of nitidine chloride and camptothecin on the growth of babesia and theileria parasites. Ticks Tick-borne Dis. 9, 1192–1193. doi: 10.1007/s00436-020-06796-z 29730263

[B117] Teel P. D. Hairgrove T. (2024). Transboundary tick and tick-borne pathogen threats to cattle. Vet. Clin. North. Am. Food. Anim. Pract. 40, 305–316. doi: 10.1016/j.cvfa.2024.01.006 38402041

[B118] Tuvshintulga B. Sivakumar T. Nugraha A. B. Ahedor B. Batmagnai E. Otgonsuren D. . (2022). Combination of clofazimine and atovaquone as a potent therapeutic regimen for the radical cure of babesia microti infection in immunocompromised hosts. J. Infect. Dis. 225, 238–242. doi: 10.1093/infdis/jiab537 34664651

[B119] Tuvshintulga B. Sivakumar T. Yokoyama N. Igarashi I. (2019). Development of unstable resistance to diminazene aceturate in Babesia bovis. Int. J. Parasitol. Drugs Drug Resist. 9, 87–92. doi: 10.1016/j.ijpddr.2019.02.001 30785049 PMC6382846

[B120] Vydyam P. Ben Mamoun C. (2026a). A standardized culture medium for comparative drug efficacy across plasmodium and babesia species. Bio-protocol 16, e5625. doi: 10.21769/bioprotoc.5625 41815843 PMC12971278

[B121] Vydyam P. Ben Mamoun C. (2026b). A standardized culture medium for comparative drug efficacy evaluation across Plasmodium and Babesia species. Bio Protoc. 16, e5625. doi: 10.21769/bioprotoc.5625 41815843 PMC12971278

[B122] Vydyam P. Chand M. Gihaz S. Renard I. Heffernan G. D. Jacobus L. R. . (2024a). *In vitro* efficacy of next-generation dihydrotriazines and biguanides against babesiosis and malaria. Antimicrob. Agents Chemother. 68, e00423-24. doi: 10.1038/s41564-023-01360-8 39136469 PMC11373198

[B123] Vydyam P. Pal A. C. Renard I. Chand M. Kumari V. Gennaro J. C. . (2024b). Tafenoquine-atovaquone combination achieves radical cure and confers sterile immunity in experimental models of human babesiosis. J. Infect. Dis. 229, 161–172. doi: 10.1093/infdis/jiad315 38169301 PMC10786256

[B124] Woodland J. G. Hunter R. Smith P. J. Egan T. J. (2018). Chemical proteomics and super-resolution imaging reveal that chloroquine interacts with plasmodium falciparum multidrug resistance-associated protein and lipids. ACS Chem. Biol. 13, 2939–2948. doi: 10.1021/acschembio.8b00583 30208272

[B125] Wu J. Li Y. He Q. Yang X. (2023). Exploration of the use of natural compounds in combination with chemotherapy drugs. Molecules 28, 1022. doi: 10.3390/molecules28031022 36770689 PMC9920618

[B126] Yin M. Zhang H. B. Tao Y. Yao J. M. Liu H. Win H. H. . (2022). Optimization of an evaluation method for anti-babesia microti drug efficacy. Acta Trop. 225, 106179. doi: 10.1016/j.actatropica.2021.106179 34627758

[B127] Yu L. Liu Q. Luo W. Zhao J. Alzan H. F. He L. (2022). The structural basis of babesia orientalis lactate dehydrogenase. Front. Cell. Infect. Microbiol. 11, 790101. doi: 10.1126/science.aaf4417 35071043 PMC8766848

[B128] Yu J. Yang H. Wang J. Chen S. Huang Z. Wang J. . (2024). Effects of gossypol acetate on growth, serum biochemical parameters, and intestinal health of goslings. Poult. Sci. 103, 104025. doi: 10.1016/j.psj.2024.104025 39003791 PMC11298947

[B129] Yu L. Zhan X. Liu Q. Sun Y. Li M. Zhao Y. . (2020). Identifying the naphthalene-based compound 3,5-dihydroxy 2-napthoic acid as a novel lead compound for designing lactate dehydrogenase-specific antibabesial drug. Front. Pharmacol. 10, 1663. doi: 10.3389/fphar.2019.01663 32116673 PMC7025647

[B130] Yuan Y. Shi C. Zhao H. (2023). Machine learning-enabled genome mining and bioactivity prediction of natural products. ACS Synth. Biol. 12, 2650–2662. doi: 10.1021/acssynbio.3c00234 37607352 PMC10615616

[B131] Zaugg J. L. Lane V. M. (1989). Evaluations of buparvaquone as a treatment for equine babesiosis (babesia equi). Am. J. Vet. Res. 50, 782–785. doi: 10.2460/ajvr.1989.50.05.782 2729726

[B132] Zhai X. Li L. Zhang P. Guo Y. Jiang H. He W. . (2022). Evaluation of the inhibitory effects of six natural product extracts against babesia gibsoni *in vitro* and *in vivo*. J. Parasitol. 108 (4), 301–305. doi: 10.1645/21-108 35877154

[B133] Zhang Y. Alvarez-Manzo H. Leone J. Schweig S. Zhang Y. (2021). Botanical medicines cryptolepis sanguinolenta, artemisia annua, scutellaria baicalensis, polygonum cuspidatum, and alchornea cordifolia demonstrate inhibitory activity against babesia duncani. Front. Cell. Infect. Microbiol. 11, 624745. doi: 10.1097/im9.0000000000000069 33763384 PMC7982592

[B134] Zhang L. Song J. Kong L. Yuan T. Li W. Zhang W. . (2020). The strategies and techniques of drug discovery from natural products. Pharmacol. Ther. 216, 107686. doi: 10.1016/j.pharmthera.2020.107686 32961262

[B135] Zheng J. Cao Z. Yousaf W. Haseeb A. Wang Z. Wang H. (2025). Ellagic acid mitigates rotavirus-induced intestinal injury via bidirectional "immune-microbiota" regulatory effect. Front. Cell. Infect. Microbiol. 15, 1686918. doi: 10.3389/fcimb.2025.1686918 41532082 PMC12791041

