# Supplementary material for: Natural products against Babesiosis: active compounds, molecular mechanisms, application limitations and translational development
Source: Front Cell Infect Microbiol. 2026 Aug 24;16:1921065. doi: 10.3389/fcimb.2026.1921065 (PMC13548037; doi:10.3389/fcimb.2026.1921065)
Supplement: Supplementary file 1 [file Table1.docx]

| **Parasite Species** | **Susceptible Animals** | **Transmission Vectors** | **Epidemiological Status** | **Ref.** |
| --- | --- | --- | --- | --- |
| *B. microti* | Humans, Microtus, Mice | *Ixodes scapularis* | North America: northeast and midwest  Europe: Germany and Poland  Asia: southwest China and Japan | [7-9] |
| *B. duncani* | Human, Odocoileus hemionus | *Dermacentor albipictus* | North America: west coast | [5,10] |
| *B. divergens* | Human, Cattle | *Ixodes ricinus* | Europe: France and Ireland  America | [11-13] |
| *B. bovis* | Human, Cattle | *Rhipicephalus microplus* and *Rhipicephalus annulatus* | Asia: Northwest China  South America: Central part | [14,15] |
| *B. bigmina* | Human, Cattle | *Rhipicephalus microplus* | South America: middle part  Asia: southwest China | [15,16] |
| *B. canis* | Dog | *Dermacentor reticulatus* | Europe | [17] |
| *B. gibsoni* | Dog | *Haemaphysalis longicornis* | Asia: Southeast Asia  North America: Central part | [17] |

**Table 1 Global distribution and epidemic areas of major zoonotic Babesia species**
